# The efficacy of psychological treatments on body dysmorphic disorder: a meta-analysis and trial sequential analysis of randomized controlled trials

**DOI:** 10.1017/S0033291724002733

**Published:** 2024-11

**Authors:** Yinong Liu, Lizu Lai, Sabine Wilhelm, Katharine A. Phillips, Yunxiao Guo, Jennifer L. Greenberg, Zhihong Ren

**Affiliations:** 1 Key Laboratory of Adolescent Cyberpsychology And Behavior (Ministry Of Education), Key Laboratory of Human Development and Mental Health Of Hubei Province, School of Psychology, Central China Normal University, Wuhan, China; 2 Department of Psychiatry, Massachusetts General Hospital and Harvard Medical School, Boston, MA, USA; 3 New York-Presbyterian Hospital and Weill Cornell Medical College, New York, NY, USA

**Keywords:** body dysmorphic disorder, meta-analysis, psychological treatment, randomized controlled trials, trial sequential analysis

## Abstract

This meta-analysis and trial sequential analysis (TSA) of randomized controlled trials (RCTs) on the psychological treatment of body dysmorphic disorder (BDD) was conducted to evaluate the intervention effects and robustness of the evidence. This study included 15 RCTs up until 15 June 2024, with 905 participants. Results showed significant improvements in BDD symptoms (*g* = −0.97), depression (*g* = −0.51), anxiety (*g* = −0.72), insight/delusion (*g* = −0.57), psychosocial functioning (*g* = 0.45), and quality of life (*g* = 0.44), with effects sustained from 1 to 6 months follow-up. RCTs with a waitlist/inactive control reported larger effect sizes for post-intervention BDD symptoms compared to those with a placebo/active control group. In addition, studies with low risk of bias demonstrate larger effect sizes for post-intervention psychosocial functioning compared to studies with some concerns. Notably, the presence of exposure and response prevention in the treatment, as well as the mode of delivery (face-to-face or digital), did not have a significant impact on the intervention outcomes. Females exhibited greater effect sizes in post-intervention BDD symptoms and psychosocial functioning than males. With increasing age, the effect size for insight/delusion symptoms diminished. Longer session duration was associated with larger effect sizes for BDD symptoms, depression at post-treatment, and depression at follow-up. TSA indicated robust evidence for depression at post-treatment and BDD symptoms, while the remaining outcome variables did not meet the desired level of evidence. In conclusion, this study underscores the effectiveness of psychological treatments in reducing BDD symptoms and improving related outcomes, highlighting the need for further research to confirm the impact of these therapies on other outcomes.

## Introduction

Body dysmorphic disorder (BDD) is a distressing and often-disabling mental disorder characterized by distressing or impairing preoccupation with nonexistent or slight defects in one's physical appearance (American Psychiatric Association, 2022). Despite its prevalence, BDD is frequently misdiagnosed or undiagnosed (Schulte, Schulz, Wilhelm, & Buhlmann, 2020). Epidemiological studies indicate that the point prevalence of BDD in the general population ranges from 1.7% to 2.9% (Buhlmann et al., 2010; Koran, Abujaoude, Large, & Serpe, 2008; Rief, Buhlmann, Wilhelm, Borkenhagen, & Brähler, 2006; Schieber, Kollei, de Zwaan, & Martin, 2015).

BDD is associated with marked functional impairment, diminished quality of life (Phillips, Menard, Fay, & Pagano, 2005), delusional thinking (Phillips, 2004), high rates of comorbid disorders, and an elevated risk of suicidal ideation and behavior in the absence of appropriate treatment (Angelakis, Gooding, & Panagioti, 2016; Gunstad & Phillips, 2003). Therefore, effective treatments are imperative to address the disorder and alleviate its adverse consequences.

Since 1995, a number of studies have demonstrated the effectiveness of cognitive-behavioral therapy (CBT) and behavior therapy (BT) for BDD (Campisi, 1995; McKay et al., 1997; Rosen, Reiter, & Orosan, 1995; Wilhelm, Otto, Lohr, & Deckersbach, 1999). According to the National Institute for Health and Clinical Excellence (NICE, 2006), CBT, including exposure and response prevention (ERP), is recommended as the first-line treatment for children and adolescents with BDD. For adult patients, NICE recommends either CBT (including ERP) or the combination of CBT with selective serotonin reuptake inhibitors (SSRIs) as treatment options. Over the past decade, various other psychological treatments have been developed for BDD, including emotion-focused transdiagnostic treatment (Mohajerin, Bakhtiyar, Olesnycky, Dolatshahi, & Motabi, 2019), metacognitive therapy without ERP (Rabiei, Mulkens, Kalantari, Molavi, & Bahrami, 2012), short-term interpretive bias modification therapy (CBM-I) (Summers & Cougle, 2016; Wilver & Cougle, 2019), as well as mindfulness-based therapy like acceptance and commitment therapy (ACT) (Pickard, Lumby, & Deane, 2021), and mindfulness-based cognitive therapy (MBCT) (Gu & Zhu, 2023). At the same time, an increasing number of researchers have explored the efficacy of digital psychological treatment for BDD (Enander et al., 2016; Summers & Cougle, 2016; Wilhelm et al., 2022; Wilver & Cougle, 2019).

A previous meta-analysis, which included 15 eligible studies (including 2 randomized controlled trials [RCTs] of psychological treatment), found no significant difference in efficacy between BT and CBT for BDD (Williams, Hadjistavropoulos, & Sharpe, 2006). Ipser, Sander, and Stein (2009) confirmed the efficacy of psychological treatment for BDD in a meta-analysis that included only two RCTs with available data. Similarly, a meta-analysis conducted by Harrison, Fernández de la Cruz, Enander, Radua, and Mataix-Cols (2016), incorporating seven RCTs, demonstrated that CBT significantly alleviated BDD and depressive symptoms and improved BDD-related insight/delusionality in individuals with BDD compared to waitlist and psychological placebo groups. In addition, the reduction in BDD symptoms was sustained over a 2–4 months of follow-up period. In the same year, a systematic qualitative review identified CBT, metacognitive therapy, and SSRIs as beneficial treatments, based on five RCTs of psychological treatments and three RCTs of pharmacological treatment (Phillipou, Rossell, Wilding, & Castle, 2016).

However, previous meta-analyses have some limitations. In recent years, there has been a growing number of RCTs investigating psychological treatments for BDD (Gu & Zhu, 2023; Mohajerin et al., 2019; Ritter, Schüller, Berkmann, von Soosten-Höllings-Lilge, & Stangier, 2023; Torkian, Zanjani, Pourkmali, & Omidi, 2022; Wilhelm et al., 2019, 2022; Wilver & Cougle, 2019). Nevertheless, previous meta-analyses included only a limited number of RCTs with small sample sizes, and the robustness of the evidence from these meta-analysis results was not adequately assessed. Notably, some meta-analysis focused exclusively on CBT. In contrast, our meta-analysis includes a broader range of psychological treatments, offering a more comprehensive evaluation of therapeutic options for BDD. Meta-analyses with a limited number of trials are susceptible to type I errors (overestimation of effect sizes) or type II errors (underestimation of effect sizes) and possess low credibility (Pereira & Ioannidis, 2011). In meta-analyses with a limited number of trials, trial sequential analysis (TSA) is recommended to control spurious errors and establish the reliability of the evidence (Wetterslev, Thorlund, Brok, & Gluud, 2008, 2017). Thus, it is necessary to conduct a comprehensive updated meta-analysis with TSA based on RCTs of psychological treatment for BDD. Additionally, while previous research has explored both predictor and moderator variables in the context of CBT for BDD, findings regarding predictors of treatment outcomes have been inconsistent, and research on moderators remains limited (Flygare et al., 2020; Malcolm, Pikoos, Castle, & Rossell, 2021; Phillips et al., 2021; Rautio et al., 2022).

In light of these considerations, we have undertaken a new meta-analysis and TSA of psychological treatment for BDD. Our objectives are threefold: First, to evaluate the immediate and long-term effects of psychological treatment for patients with BDD. Second, to ascertain whether demographic characteristics, comorbidities, current SSRIs use, and intervention characteristics impact effect sizes. Finally, to confirm the robustness of the evidence presented in the meta-analysis.

## Method

This meta-analysis adhered to the Preferred Reporting Items for Systematic Reviews and Meta-Analysis (PRISMA) standards, and the protocol for the study has been registered with PROSPERO (CRD 42023420253).

### Literature search

Two independent reviewers (Y. L. and Y. G.) conducted an initial systematic literature search on 6 September 2023, and update the search on 15 June 2024. We systematically searched the published and unpublished literature in following databases: Web of Science, PsycINFO, PubMed, Scopus, Cochrane Library, Proquest, Embase, and Europe PMC. Our search strategy utilized a combination of keywords related to BDD and RCTs (see Appendix B). No restrictions were imposed on keywords related to psychological treatment to ensure a comprehensive collection of RCTs related to psychological treatment of BDD. Additionally, relevant reviews, meta-analyses, and reference lists of included articles were manually screened.

### Study selection

Studies were eligible if they: (a) were RCTs; (b) investigated the effects of psychological treatments; (c) included a control group (waitlist control, no-treatment control, treatment-as-usual control, active control, or psychological placebo control); (d) involved participants who met diagnostic criteria for BDD as defined by any version of the Diagnostic and Statistical Manual of Mental Disorders (DSM) (including DSM-III-R, DSM-IV/DSM-IV-TR, and DSM-5/DSM-5-TR); (e) included measurements of BDD symptoms. Non-English articles were excluded from the analysis.

Two researchers (Y. L. and L. L.) independently conducted the literature screening. Abstracts were initially screened to ascertain if the studies met the eligibility requirements. Subsequently, full-text screening was conducted for the eligible articles. In cases where information within an article was insufficient to calculate effect sizes, the corresponding authors were contacted to request the necessary data. The final list of included studies was determined through discussions between Y. L., K. A. P., J. L. G., and S. W.

### Data collection process

Data extraction was conducted by Y. L. and L. L., with Y. G. performing a verification check. Any disagreements were resolved through discussion. The following information was documented for each article: study details (authors, country, publication year, and sample size), sample demographics (percentage of female patients, age), clinical characteristics (diagnostic criteria, percentage of patients with comorbid major depressive disorder, and percentage of patients using SSRIs), intervention characteristics (number of sessions, duration of session and intervention, format, delivery mode), measurement tools (all measurements for primary and secondary outcomes), and statistical data used to calculate effect sizes (sample sizes, mean, and standard deviations at post-intervention and follow-up).

### Risk of bias assessment

Risk of bias was independently conducted by Y. L. and L. L. using the revised Cochrane Risk of Bias Tool (ROB 2.0) (Sterne et al., 2019). This evaluation considered five key areas: random sequence generation, allocation concealment, outcome assessment blinding, incomplete outcome data management, and selective reporting. A third researcher was engaged to resolve any disagreements regarding the assessment of bias risk.

### Meta-analytic strategy

The meta-analysis was performed using Comprehensive Meta-Analysis (CMA) Version 3.0 software (Borenstein, Hedges, Higgins, & Rothstein, 2014). Effect sizes (Hedge's *g*) were calculated based on the mean, standard deviation, and sample size of post-test and follow-up measurements for both the psychological treatments and control groups. Hedge's *g*, a modification of Cohen's *d* that corrects for sample size-related bias, was used (Hedges & Olkin, 1985). Effect sizes of 0.2, 0.5, and 0.8 were considered small, medium, and large, respectively (Borenstein, Cooper, Hedges, & Valentine, 2009). A random-effects model was employed to account for the risk of type I error by combining effect sizes (Berkeljon & Baldwin, 2009).

The *Q* test and *I*^2^ were used for heterogeneity testing, with *I*^2^ representing the proportion of total variation due to between-study variability (*I*^2^ = 25%, 50%, 75%: low, moderate, high heterogeneity, respectively). When the *Q* test was significant and *I*^2^ exceeded 75%, heterogeneity among the studies was indicated, justifying the use of a random-effects model (Huedo-Medina, Sánchez-Meca, Marín-Martínez, & Botella, 2006).

### Outlier and influence analyses

Outlier and influence analyses were conducted using the metafor package within the R environment (Viechtbauer & Viechtbauer, 2015). Studentized deleted residual (SDR) values exceeding 1.96 were considered indicative of outlier effect sizes (Shi, Ren, Zhao, Zhang, & Chan, 2021; Viechtbauer & Cheung, 2010). Influence indicators were evaluated through Cook's distance (CD) and DFBETAS values. A CD value greater than 0.45 (Weisberg & Cook, 1982) or a DFBETAS value greater than 1 (Lai, Liu, McCracken, Li, & Ren, 2023; Viechtbauer & Cheung, 2010) suggested that an effect size significantly influenced the overall effect size.

### Publication bias

Publication bias was assessed by examining the funnel plot and conducting Egger's test of the intercept to quantify and test the significance of bias captured by the funnel plot (Bowden, Davey Smith, & Burgess, 2015). The Egger's regression test (Egger, Smith, Schneider, & Minder, 1997) was performed to formally assess the statistical significance of the funnel plot asymmetry.

### Moderator analysis

Subgroup analyses were conducted for categorical moderator variables, including the method of treatment delivery, risk of bias, type of treatment, and control group. Additionally, meta-regression analyses were performed for continuous moderator variables, including the percentage of female patients, average age, percentage of patients with comorbid major depressive disorder, percentage of patients using SSRIs, number of sessions, duration of each session, and total duration of the intervention. Subgroup analyses required a minimum of three studies for each analysis, following the guidelines of van Eldik et al. (2020), while meta-regression analyses necessitated a minimum of six studies for each analysis, as recommended by Du, Witthöft, Zhang, Shi, and Ren (2023).

### Trial sequential analysis

To mitigate the risk of false-positive results in the meta-analysis (type I error), which can occur due to repeated significance tests or misinterpretation of random errors, TSA was employed (Wetterslev et al., 2008, 2017). TSA software version 0.9.5.10 Beta (Thorlund et al., 2017) was used to conduct TSA studies based on the included investigations. A cumulative *Z*-curve was calculated and contrasted with an adjusted *Z*-curve established according to predetermined monitoring criteria. TSA also calculated the required information size (RIS), representing the minimum number of participants needed to detect a specific intervention effect in a meta-analysis. The alpha (type I error) level was set at 5%, and the traditional significance limit was defined within the interval of ±1.96 *Z* value in the standard normal distribution. We set statistical power at 90% (Thorlund et al., 2017).

## Results

### Study selection and characteristics

**Figure 1.**
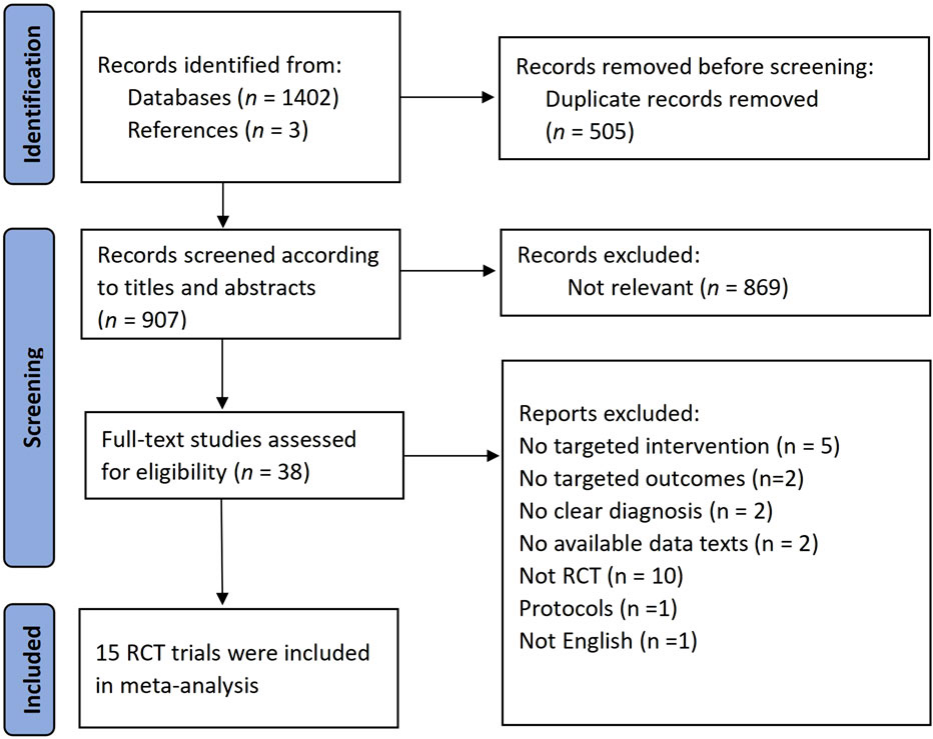
PRISMA diagram showing the results of the literature search.

Figure 1 illustrates the study selection procedure. A total of 15 RCTs with 905 participants diagnosed with BDD, were included in the meta-analysis. The majority of the participants in these studies were adults, with only one study focusing on adolescents. The average age of the participants was 29.53 years (s.d. = 9.72), with 76.9% (range = 51.6–100) of them being female. On average, 28.26% of patients had current comorbid major depressive disorder (range = 10–54.3), and approximately of 20% used SSRI during treatment (range = 0–47.65). Nine studies tested an ERP-based variation of CBT for BDD (Enander et al., 2016; Mataix-Cols et al., 2015; Mohajerin et al., 2019; Rosen et al., 1995; Veale et al., 1996, 2014; Wilhelm et al., 2014, 2019, 2022). Two studies exclusively examined cognitive therapy (Rabiei et al., 2012; Ritter et al., 2023), and two studies investigated mindfulness-based therapies, such as ACT (Torkian et al., 2022) and MBCT (Gu & Zhu, 2023). Furthermore, two studies focused on interpretation bias modification (Summers & Cougle, 2016; Wilver & Cougle, 2019). The average treatment length was 12.8 weeks (range = 2–36), with an average of 12.29 sessions (range = 4–22). The average session duration was 65.63 min (range = 15–120). Eight studies had a control group with a waitlist, no-treatment, or treat-as-usual condition (waitlist/inactive control), while seven studies had a control group with an active control group (any credible psychological intervention that includes only non-specific components of therapy, such as anxiety management, psychoeducation, supportive therapy, or relaxation) or a psychological placebo group (sham training or only offered information associated with BDD). Twelve of the 15 studies conducted follow-up assessments ranging from 1 to 6 months, with nine comparing the psychological treatment with the control group.

Six studies were conducted in the United States, three in the United Kingdom, three in Iran, and one each in China, Germany, and Sweden, respectively. Details for each study are provided in Table 1. One paper in another language was found in the literature search and excluded (Habibollahi & Soltanizadeh, 2016). All but one study (Torkian et al., 2022) were peer reviewed. The results of the study quality assessments are shown in Appendix C. One study was identified as high risk due to the use of self-report BDD-YBOCS with only first 10 items. While seven studies raised some concerns related to at least one of the following factors: the randomization process, missing outcome data, or measurement of the outcome. And seven studies were considered low-risk.

**Table 1.** Characteristics of included studies

Study (year)	Country	Sample size	Age mean ± s.d.	Female (%)	Comorbid major depressive disorder (%)	Current SSRIs (%)	BDD symptom	Secondary outcomes	Intervention	Control group	Number of sessions	Duration of sessions (min)	Duration of treatment (week)	Delivery
Enander et al. (2016)	Sweden	94	28.82 ± 4.78	85.1	54.3	13.83	BDD-YBOCS	Depression: MADRS-S	CBT, including ERP	Supportive therapy	8	–	12	Internet-based
								Quality of life: EQ5D						
								Psychosocial functioning: GAF						
Gu and Zhu (2023)	China	116	32.5 ± 8.5	76.7	–	10.34	BDD-YBOCS	Depression: BDI-II	MBCT	TAU	8	90	8	Face-to-face
								Anxiety: BAI						
								Insight: BABS						
Mataix-Cols et al. (2015)	United Kingdom	30	16 ± 1.7	86.7	23.3	16.67	BDD-YBOCS-A	Depression: BDI-Y	CBT, including ERP	Psychoeducation	14	60/90	16	Face-to-face
								Quality of life: BIQLI						
								Psychosocial functioning: CGAS						
								Insight: BABS						
Mohajerin et al. (2019)	Iran	128	28.85 ± 6.07	51.6	19.5	47.65	BDD-YBOCS	Depression: BDI-II	UP, including ERP	Waitlist/TAU	14	60	20	Face-to-face
								Insight: BABS						

Rabiei et al. (2012)	Iran	20	25.2 ± 6.5	90	10	0	BDD-YBOCS	–	MCT	Waitlist	8	45-60	8	Face-to-face
Ritter et al. (2023)	Germany	40	27.93 ± 8.34	67.5	20	27.5	BDD-YBOCS	Depression: BDI-II	CT	Waitlist	20	50/100	36	Face-to-face
								Quality of life: EUROHIS-QoL						
								Psychosocial functioning: GAF						
								Insight: BABS						
Rosen et al. (1995)	United States	54	36.5 ± 9.5	100	–	–	BDDE	–	CBT, including ERP	No treatment	8	120	12	Face-to-face
Summers and Cougle (2016)	United States	38	19.79 ± 2.9	80	–	18.42	BDD-YBOCS	Depression: DASS-depression	CBM-I	Placebo control	4	30	2	Computer-based
								Anxiety: DASS-anxiety						
Torkian et al. (2022)	Iran	34	22.65 ± 2.94	61.8	–	0	BDD-YBOCS	Depression: DASS-depression	ACT	Placebo control	8	90	8	Internet-based
								Anxiety: DASS-anxiety						
Veale et al. (2014)	United Kingdom	46	30 ± 8.52	58.7	–	45.7	BDD-YBOCS	Depression: PHQ-9	CBT, including ERP	Anxiety management	12 (16)	60	12	Face-to-face
								Anxiety: GAD-7						
								Quality of life: BIQLI						
								Insight: BABS						
Veale et al. (1996)	United Kingdom	19	35.36 ± 10.63	90	–	–	BDD-YBOCS,BDDE	Depression: HADS-D	CBT, including ERP	Waitlist	12	–	12	Face-to-face
								Anxiety: HADS-A						
Wilhelm et al. (2019)	United States	120	33.94 ± 13.02	76.7	32.5	25.8	BDD-YBOCS	Depression: BDI-II	CBT, including ERP	Supportive Psychotherapy	22	60	18	Face-to-face
								Quality of life: Q-LES-Q-SF						
								Psychosocial functioning: SDS						
								Insight: BABS						
Wilhelm et al. (2014)	United States	36	34.84 ± 11.55	61	44.4	–	BDD-YBOCS	Depression: BDI	CBT, including ERP	Waitlist	12 (22)	60	12 (24)	Face-to-face
								Psychosocial functioning: SDS						
								Insight: BABS						
Wilhelm et al. (2022)	United States	80	27 ± 9.67	83.75	26.3	25	BDD-YBOCS	Depression: QIDS-SR	CBT, including ERP	Waitlist	–	–	12	Smartphone app-based
								Quality of life: Q-LES-Q-SF						
								Psychosocial functioning: SDS						
								Insight: BABS						
Wilver and Cougle (2019)	United States	50	28.52 ± 9.32	84	24	10	BDD-YBOCS	Depression: BDI-II	CBM-I	PMR	8	15	4	Internet-based
								Anxiety: BAI						
								Insight: BABS						

SSRIs, selective serotonin reuptake inhibitors; BDD, body dysmorphic disorder; BDD-YBOCS, Modified Yale Brown Obsessive Compulsive Scale for Body Dysmorphic Disorder; BDDE, Body Dysmorphic Disorder Examination; MADRS, Montgomery and Asberg Depression Rating Scale; EQ5D, Visual Analogue Scale of Euroqol; GAF, Global Assessment of Functioning scale; BDI, Beck Depression Inventory; BAI, Beck Anxiety Inventory; BABS, Brown Assessment of Beliefs Scale; BIQLI, Body Image Quality of Life Inventory; CGAS, Children's Global Assessment Scale; EUROHIS-QoL, European Health Interview Survey-Quality of Life; DASS, Depression Anxiety Stress Scales; PHQ-9, Patient Health Questionnaire-9; GAD-7, Generalised Anxiety Disorder-7; HADS, Hospital Anxiety and Depression Scale; Q-LES-Q-SF, Quality of Life Enjoyment and Satisfaction Questionnaire-Short Form for quality of life; QIDS-SR, Quick Inventory of Depressive Symptomatology – Self Report; SDS, Sheehan Disability Scale; CBT, cognitive behavioral therapy; ERP, exposure and response prevention; MBCT, mindfulness-based cognitive therapy; UP, Unified Protocol for the Transdiagnostic Treatment of Emotional Disorders; MCT, metacognitive therapy; CT, cognitive therapy; ACT, acceptance and commitment therapy; CBM-I, Cognitive Bias Modification of Interpretation; PMR, Progressive Muscle Relaxation; TAU, treatment-as-usual.

### The efficacy of psychological treatments on BDD

Table 2 presents the effects of psychological treatments on BDD symptoms, depression, anxiety, BDD-related insight/delusionality, psychosocial functioning, and quality of life (forest plot provided in the Appendix D). Large effect sizes were observed for BDD symptoms, depression, and BDD-related insight/delusionality at post-treatment (BDD symptoms: *g* = −1.47, 95% CI [−2.11 to −0.84], *p* < 0.001; depression: *g* = −1.16, 95% CI [−1.24 to −0.69], *p* < 0.001; BDD-related insight/delusionality: 95% CI [−2.77 to −0.64], *p* < 0.001) and at follow-up (BDD symptoms: *g* = −1.59, 95% CI [−2.59 to −0.59], *p* < 0.01; depression: *g* = −1.27, 95% CI [−2.23 to −0.15], *p* < 0.05; BDD-related insight/delusionality: *g* = −2.43, 95% CI [−4.13 to −0.73], *p* < 0.01). Medium effect sizes were found for anxiety at post-treatment (*g* = −0.50, 95% CI [−0.88 to −0.14], *p* < 0.01), but were not significant at follow-up (*g* = −0.43, 95% CI [−0.89 to 0.04], *p* > 0.05). The results also indicated that psychological treatment significantly improved the level of psychosocial functioning and quality of life at post-treatment (psychosocial functioning: *g* = 0.45, 95% CI [0.17 to 0.74], *p* < 0.01; quality of life: *g* = 0.44, 95% CI [0.17 to 0.71], *p* < 0.001) and at follow-up (psychosocial functioning: *g* = 0.53, 95% CI [0.18 to 0.89], *p* < 0.001; quality of life: *g* = 0.36, 95% CI [0.02 to 0.69], *p* < 0.05), although effect sizes were smaller for psychosocial functioning and quality of life than for symptom variables.

**Table 2.** Estimated pooled effect sizes for psychological treatment on body dysmorphic disorder

Time	Outcomes	Sample size		Heterogeneity		Effect size		Egger's regression test	
		*k*	*n*	*Q*	*I*^2^ (%)	Hedge's *g*	95% CI	Intercept	*t*
Post-treatment	Body dysmorphic disorder	15	831	223.98***	93.75	−1.47***	(−2.11 to −0.84)	–	–
	Body dysmorphic disorder^a^	14	703	37.87***	65.67	−0.97***	(−1.24 to −0.69)	−1.93	1.19
	Depression	13	751	225.58***	94.68	−1.16***	(−1.86 to −0.46)	–	–
	Depression^a^	12	623	17.18	35.96	−0.51***	(−0.72 to −0.31)	−0.74	0.54
	BDD-related insight/delusionality	9	571	240.08***	96.67	−1.71**	(−2.77 to −0.64)	–	–
	BDD-related insight/delusionality^a^	8	443	19.91**	64.84	−0.57**	(−0.90 to −0.23)	−2.73	1.23
	Quality of life	6	338	7.354	32.01	0.44***	(0.17 to 0.71)	2.00	1.01
	Psychosocial functioning	6	332	8.00	37.49	0.45**	(0.17 to 0.74)	−0.81	0.35
	Anxiety	6	288	11.18*	55.27	−0.51**	(−0.88 to −0.14)	–	–
	Anxiety^b^	5	239	2.30	0.00	−0.72***	(−0.98 to −0.46)	2.02	2.43
Follow-up	Body dysmorphic disorder	9	544	199.47***	95.99	−1.59**	(−2.59 to −0.59)	–	–
	Body dysmorphic disorder^a^	8	416	10.62	34.10	−0.73***	(−0.98 to −0.48)	−1.22	0.73
	Depression	8	509	210.61***	96.67	−1.27*	(−2.39 to −0.15)	–	–
	Depression^a^	7	381	13.33*	54.99	−0.39*	(−0.70 to −0.08)	2.56	1.03
	BDD-related insight/delusionality	6	409	234.98***	97.87	−2.43**	(−4.13 to −0.73)	–	–
	BDD-related insight/delusionality^a^	5	281	14.84**	73.05	−0.55*	(−1.03 to −0.08)	−0.87	0.20
	Quality of life	4	208	2.22	0.00	0.36*	(0.02 to 0.69)	−0.19	0.06
	Psychosocial functioning	3	179	2.70	25.79	0.53**	(0.18 to 0.89)	1.22	0.46
	Anxiety	4	207	7.80*	61.55	−0.43	(−0.89 to 0.04)	3.47	0.95
	Anxiety^b^	3	163	2.48	19.29	−0.65***	(−1.01 to −0.29)	2.56	1.01

*Note*: *p < 0.05, **p < 0.01, ***p < 0.001; *k* = the number of trials; *n* = the number of effect sizes; for BDD symptoms, depression, BDD-related insight/delusionality, and anxiety, a negative *g* indicates a more favorable treatment outcome. Conversely, for psychosocial functioning and quality of life, a negative *g* indicates a worse outcome.

a Exclude outlier Mohajerin et al. (2019).

b Exclude outlier Wilver and Cougle (2019).

The results of heterogeneity tests were significant for BDD symptoms, depression, BDD-related insight/delusionality, and anxiety at both post-treatment and follow-up (significant *Q* value and *I*^2^ > 50%), indicating that moderation tests are necessary.

### Outlier and influence

The results of outlier and influence analyses are illustrated in Appendix E. In the study by Mohajerin et al. (2019), the outcomes for BDD, depression, and BDD-related insight/delusionality exceeded the cut-off values for SDR, CDs, and DFBETASs (SDR > 1.96, CDs > 0.45, DFBETAS > 1). Furthermore, the outcome for anxiety in the study conducted by Wilver and Cougle (2019) also surpassed the threshold values for three indicators. This suggests that these studies were influential outliers in the mentioned outcomes. Therefore, these studies were excluded from the analysis of these specific outcomes.

The analysis results after excluding these outliers are presented in Table 2. After removing outlier studies, the effect sizes for BDD symptom severity, depression, and BDD-related insight/delusionality reduced but remained statistically significant at post-test (BDD symptoms: *g* = −0.97, 95% CI [−1.24 to −0.69], *p* < 0.001; depression: *g* = −0.51, 95% CI [−0.72 to −0.31], *p* < 0.001; BDD-related insight/delusionality: *g* = −0.57, 95% CI [−0.90 to −0.23], *p* < 0.001) and at follow-up assessments (BDD symptoms: *g* = −0.73, 95% CI [−0.98 to −0.48], *p* < 0.001; depression: *g* = −0.39, 95% CI [−0.70 to −0.08], *p* < 0.05; BDD-related insight/delusionality: *g* = −0.55, 95% CI [−1.03 to −0.08], *p* < 0.05). However, the effect size for anxiety increased at post-treatment (*g* = −0.72, 95% CI [−0.98 to −0.46], *p* < 0.001) and at follow-up (*g* = −0.65, 95% CI [−1.01 to −0.29], *p* < 0.001), with the effect becoming significant at follow-up. Furthermore, after removing outlier studies, the heterogeneity of BDD symptoms, depression, BDD-related insight/delusionality, and anxiety decreased.

To ensure the stability of the results, in the results that follow (publication bias, moderator analyses, and TSA) we exclude the previously noted influential outlier studies from analyses of the mentioned outcomes.

### Publication bias

The funnel plots for the outcomes can be found in Appendix F. Based on the funnel plot and the results of the Egger regression test, no potential publication bias was observed across all outcome variables.

It is important to note that funnel plots and Egger's test are not recommended for detecting publication bias when the number of included studies is less than 10 (Egger et al., 1997). Therefore, the funnel plot results for BDD-related insight/delusionality, quality of life, anxiety, psychosocial functioning at post, and all outcomes at follow-up are provided for reference purposes only and should be interpreted with caution.

### Moderator

The results of subgroup analyses are displayed in Table 3. Subgroup analysis indicated that, control group type was a significant moderator for the efficacy of psychological treatment on BDD symptoms at post-treatment; studies with a waitlist/inactive control demonstrated larger effects compared to studies with a placebo/active control (*p*_between_ < 0.05). In addition, study quality significantly moderates the efficacy of psychological treatment on psychosocial functioning at post-treatment; studies with low risk of bias demonstrate larger effect sizes compared to studies with some concerns (*p*_between_ < 0.05). There was no significant difference in effects between digitally based interventions and face-to-face interventions across all outcome variables. In addition, no significant differences were found in the short-term and long-term effectiveness of psychological treatments that included ERP compared to those that did not include ERP for any of the outcomes.

**Table 3.** Subgroup analyses examining moderators of psychological treatment on body dysmorphic disorder

Time	Subgroup	*k*	*g*	95% CI	*p*	*Q*	*I*^2^ (%)	*Q*_subgroup_	*p*_subgroup_
BDD symptoms at post-treatment	**Type of control group**							3.95	**0**.047*
	Active/placebo	7	−0.71	(−0.98 to −0.45)	0.000	9.23	35.03		
	Inactive/waiting-list	7	−1.25	(−1.71 to −0.79)	0.000	19.96	69.94		
	**Type of treatment**							2.52	0.112
	Including ERP	8	−1.15	(−1.55 to −0.74)	0.000	25.25	72.28		
	Without ERP	6	−0.72	(−1.05 to −0.39)	0.000	8.40	40.68		
	**Delivery**							0.77	0.379
	Face-to-face	9	−1.07	(−1.45 to −0.70)	0.000	23.94	66.59		
	Digitally based	5	−0.81	(−1.26 to −0.35)	0.000	13.26	69.83		
	**Risk of bias**							0.38	0.54
	Low	6	−0.89	(−1.25 to −0.53)	0.000	13.61	63.27		
	Some concerns	7	−1.09	(−1.62 to −0.56)	0.000	24.09	75.09		
BDD symptoms at follow-up	**Type of treatment**							0.03	0.87
	Including ERP	4	−0.75	(−1.04 to −0.46)	0.000	3.43	12.59		
	Without ERP	4	−0.70	(−1.19 to −0.22)	0.005	7.13	57.89		
	**Delivery**							0.55	0.46
	Face-to-face	5	−0.81	(−1.09 to −0.52)	0.000	4.76	15.94		
	Digitally based	3	−0.57	(−1.11 to −0.05)	0.033	5.11	60.84		
Depression at post-treatment	**Type of control group**							1.09	0.30
	Active/placebo	7	−0.41	(−0.61 to −0.21)	0.000	5.77	0.00		
	Inactive/waiting-list	5	−0.66	(−1.07 to −0.24)	0.002	9.00	55.58		
	**Type of treatment**							0.23	0.64
	Including ERP	7	−0.56	(−0.85 to −0.27)	0.000	10.30	41.73		
	Without ERP	5	−0.45	(−0.79 to −0.12)	0.008	6.82	41.36		
	**Delivery**							0.79	0.37
	Face-to-face	7	−0.60	(−0.91 to −0.30)	0.000	10.61	43.47		
	Digitally based	5	−0.41	(−0.69 to −0.13)	0.004	5.42	26.21		
	**Risk of bias**							0.001	0.98
	Low	6	−0.55	(−0.76 to −0.35)	0.000	7.83	36.18		
	Some concerns	5	−0.48	(−0.76 to −0.20)	0.001	8.63	53.62		
Depression at follow-up	**Type of treatment**							0.19	0.66
	Including ERP	4	−0.45	(−0.80 to −0.11)	0.010	4.33	30.75		
	Without ERP	3	−0.28	(−0.96 to 0.41)	0.428	8.99	77.76		
	**Delivery**							1.05	0.31
	Face-to-face	4	−0.54	(−0.96 to −0.11)	0.014	6.89	56.44		
	Digitally based	3	−0.20	(−0.68 to 0.29)	0.427	4.48	55.31		
BDD-related insight/delusionality at post-treatment	**Type of control group**							0.99	0.32
	Active/placebo	4	−0.42	(−0.97 to 0.14)	0.145	11.25	73.33		
	Inactive/waiting-list	4	−0.73	(−0.99 to −0.47)	0.000	2.75	0.00		
	**Type of treatment**							0.045	0.83
	Including ERP	5	−0.61	(−1.10 to −0.11)	0.016	14.21	71.85		
	Without ERP	3	−0.53	(−1.06 to –0.006)	0.053	5.68	64.80		
	**Risk of bias**							0.533	0.47
	Low	5	−0.67	(−1.04 to −0.31)	0.000	8.84	54.76		
	Some concerns	3	−0.39	(−1.04 to 0.26)	0.235	6.64	69.88		
Psychosocial functioning at post-treatment	**Type of control group**							0.001	0.98
	Active/placebo	3	0.434	(0.08 to 0.79)	0.016	3.108	35.643		
	Inactive/waiting-list	3	0.443	(−0.13 to 1.02)	0.129	4.779	58.152		
	**Risk of bias**							5.72	0.017*
	Low	3	0.70	(0.41 to 0.99)	0.000	0.49	0.00		
	Some concerns	3	0.17	(−0.16 to 0.49)	0.308	1.80	0.00		

*Note*: *p < 0.05, **p < 0.01, ***p < 0.001; *k* = the number of trials; for BDD symptoms, depression, BDD-related insight/delusionality, and anxiety, a negative g indicates a more favorable treatment outcome.

The results of meta-regression analysis are displayed in Table 4. Compared to males, females exhibited larger effect sizes in immediate post-intervention BDD disorder symptoms (*p* < 0.05) and psychosocial functioning (*p* < 0.05). As age increased, the effect size for BDD-related insight/delusionality symptoms decreased (*p* < 0.05). Additionally, longer sessions duration was associated with larger effects on BDD symptoms (*p* < 0.01) and depression symptoms (*p* < 0.01) at post-treatment, and on depression symptoms (*p* < 0.01) at follow-up. No significant moderating effects were found for the number of sessions, total treatment duration, comorbidity rate of major depressive disorder, or SSRIs use.

**Table 4.** Meta-regression analyses examining moderators of psychological treatment on body dysmorphic disorder

Outcome/time	Moderators	*k*	*β*	95% CI	*p*	*Q*	*I*^2^ (%)
BDD symptoms at post-treatment	Age	14	−0.01	(−0.07 to 0.04)	0.577	37.71	68.18
	Gender	14	−0.03	(−0.05 to –0.00)	0.019*	28.07	57.26
	Comorbid major depressive disorder	8	0.01	(−0.02 to 0.03)	0.678	17.67	66.04
	Current SSRIs usage	11	0.00	(−0.02 to 0.02)	0.675	16.70	46.11
	Duration of sessions	11	−0.01	(−0.02 to −0.00)	0.002**	15.01	40.04
	Number of sessions	13	0.02	(−0.04 to 0.08)	0.520	31.96	65.58
	Duration of treatment	14	0.01	(−0.13 to 0.15)	0.867	37.70	68.17
BDD symptoms at follow-up	Age	8	0.02	(−0.03 to 0.07)	0.491	9.92	39.52
	Gender	8	0.01	(−0.03 to 0.04)	0.681	10.34	41.97
	Current SSRIs usage	8	0.00	(−0.03 to 0.02)	0.819	10.59	43.36
	Duration of sessions	7	−0.01	(−0.02 to 0.00)	0.152	6.94	27.97
	Number of sessions	8	0.02	(−0.03 to 0.07)	0.462	9.38	36.01
	Duration of treatment	8	0.02	(−0.15 to 0.18)	0.846	10.40	42.31
Depression at post-treatment	Age	12	0.01	(−0.04 to 0.05)	0.790	17.1	41.53
	Gender	12	−0.01	(−0.03 to 0.01)	0.452	16.76	40.32
	Comorbid major depressive disorder	7	0.01	(−0.02 to 0.03)	0.624	8.23	39.27
	Current SSRIs usage	10	0.00	(−0.02 to 0.02)	0.956	9.12	12.30
	Duration of sessions	9	−0.01	(−0.02 to –0.00)	0.009**	4.18	0.00
	Number of sessions	11	0.00	(−0.05 to 0.04)	0.853	16.37	45.02
	Duration of treatment	12	−0.02	(−0.12 to 0.09)	0.725	17.11	41.54
Depression at follow-up	Age	7	−0.03	(−0.08 to 0.03)	0.332	10.51	52.42
	Gender	7	0.03	(−0.01 to 0.06)	0.152	9.25	45.92
	Current SSRIs usage	7	−0.01	(−0.04 to 0.02)	0.447	11.89	57.94
	Duration of sessions	6	−0.01	(−0.02 to −0.00)	0.003**	3.98	0.00
	Number of sessions	7	0.00	(−0.06 to 0.06)	0.941	11.79	57.6
	Duration of treatment	8	0.02	(−0.15 to 0.18)	0.846	10.40	42.31
BDD-related insight/delusionality at post-treatment	Age	8	0.06	(0.00 to 0.11)	0.039*	11.80	49.16
	Gender	8	0.00	(−0.04 to 0.04)	0.990	19.89	69.83
	Comorbid major depressive disorder	6	0.03	(−0.03 to 0.09)	0.321	14.90	73.16
	Current SSRIs usage	7	−0.01	(−0.04 to 0.03)	0.645	19.43	74.26
	Duration of sessions	7	−0.01	(−0.03 to 0.00)	0.164	11.76	57.5
	Number of sessions	7	0.00	(−0.07 to 0.08)	0.941	15.03	66.72
	Duration of treatment	8	−0.04	(−0.20 to 0.12)	0.600	19.89	69.83
Quality of life at post-treatment	Age	6	−0.04	(−0.09 to 0.02)	0.1726	5.03	20.44
	Gender	6	0.00	(−0.03 to 0.03)	0.9197	7.35	45.61
	Current SSRIs usage	6	0.01	(−0.02 to 0.04)	0.5005	6.42	37.71
	Duration of treatment	6	−0.09	(−0.21 to 0.03)	0.1294	4.81	16.81
Psychosocial functioning at post-treatment	Age	6	−0.04	(−0.08 to 0.01)	0.142	5.07	21.08
	Gender	6	0.03	(0.00 to 0.06)	0.036*	3.6	0.00
	Comorbid major depressive disorder	6	0.00	(−0.03 to 0.02)	0.812	8.00	49.98
	Duration of treatment	6	−0.03	(−0.18 to 0.12)	0.696	7.14	43.96

*Note*: *p < 0.05, **p < 0.01, ***p < 0.001; *k* = the number of trials; for BDD symptoms, depression, BDD-related insight/delusionality, and anxiety, a negative *β* indicates a more favorable treatment outcome. Conversely, for psychosocial functioning and quality of life, a negative *β* indicates a worse outcome.

### Trial sequence analyses

The results of trial sequence analyses are displayed in Appendix G. The cumulative *Z*-curve crossed both the monitoring boundary and RIS only for BDD symptoms at post-treatment and follow-up; and for depression symptoms at post-treatment. For insight, psychosocial functioning, and anxiety at post treatment, the cumulative *Z*-curve crossed the monitoring boundary but did not reach RIS. For quality of life at post-treatment and all outcomes at follow-up except BDD symptoms, the cumulative *Z*-curve neither crossed the monitoring boundary nor reached RIS.

## Discussion

This meta-analysis contributes to the existing research literature on psychological therapies for patients with BDD, and its findings are strengthened by the use of TSA. At both post-treatment and follow-up, our analysis revealed medium to large effect sizes for the reduction of BDD symptoms, depression, and BDD-related insight/delusionality with psychological treatment, aligning with prior research (Harrison et al., 2016). Notably, TSA results confirmed the robustness of these findings for BDD and depression symptoms immediately after treatment and for BDD symptoms at follow-up. Moreover, we observed small to moderate effect sizes for improving psychosocial functioning, quality of life, and reducing anxiety. However, according to the TSA analysis results, the sample size for other outcome variables did not reach the RIS. Further RCTs are needed in the future to investigate the impact of psychological therapy on depression at follow-up in patients with BDD, as well as its effects on BDD-related insight, anxiety, quality of life, and psychosocial functioning, both immediately after the treatment and at follow-up. It is important to keep in mind that most of the studies included in this meta-analysis examined CBT for BDD, and our findings should not be assumed to apply to types of therapy not included in this report.

The presence of influential outlier studies in BDD symptoms, depression, and BDD-related insight/delusionality variables, as indicated by the outlier and influence analyses, did not alter the main conclusions of the meta-analysis, although effect sizes were somewhat lower when these studies were excluded. This underscores the reliability and stability of our findings. Conversely, it is noteworthy that the exclusion of influential outlier studies led to a shift in the significance of the anxiety outcome at follow-up, transforming it from non-significant to significant. This change might be attributed to the study conducted by Wilver and Cougle (2019), employing progressive muscle relaxation as an active control group. Progressive muscle relaxation has been established as effective for anxiety (Acarturk, Cuijpers, van Straten, & de Graaf, 2009), possibly influencing the observed results.

Our subgroup analysis revealed that the type of control group significantly moderated the effect size at the post-intervention assessment for BDD. Specifically, studies with a waitlist/inactive control group showed a significantly larger effect size compared to those with a placebo/active control group. This is to be expected, because an active control condition, such as supportive therapy, or relaxation would be expected to have greater benefit than no treatment. Our analysis found no significant differences in the effectiveness of psychological treatments for patients with BDD whether including ERP or not. This finding mirrors that from a meta-analysis focused on psychological treatments for obsessive-compulsive disorder, which demonstrated similar effectiveness between ERP, cognitive restructuring, and a combination of both (Rosaalcazar, Sanchezmeca, Gomezconesa, & Marinmartinez, 2008). However, to confirm our finding, research is needed that randomizes participants to cognitive therapy alone *v.* ERP alone and ERP plus cognitive therapy. Furthermore, the comparable effectiveness of digitally based psychological interventions and traditional face-to-face interventions offers opportunities for cost-effective and accessible intervention strategies, particularly in regions with limited specialist resources for BDD treatment (Fu, 2020). However, from a clinical perspective, the closer clinical monitoring that occurs in face-to-face therapy seems more appropriate from a safety perspective for more highly suicidal and severely ill patients. Therefore, we recommend that future studies on digital interventions specifically measure suicidality, risk, and safety to ensure their suitability for patient groups.

Our meta-regression analysis illuminated several moderators influencing the efficacy of psychological treatments for patients with BDD. First, gender was identified as a significant moderator for several outcomes. Females showed greater post-treatment improvements in both BDD symptoms and psychosocial functioning compared to males. This finding may be influenced by the typically small number of males included in the trials. Due to the lower proportion of males in most study samples, the statistical power to detect effect sizes for males may be limited. This could potentially contribute to the observed larger effect sizes in females. Moreover, a younger age was associated with greater improvement in BDD-related insight/delusionality symptoms suggests the potential importance of early intervention. BDD typically emerges during childhood or adolescence, with a mean age of onset around 16.7 years (Bjornsson et al., 2013). Early-onset BDD is associated with greater illness severity, including higher rates of suicide attempts and comorbidities (Bjornsson et al., 2013). The reason for these findings is unclear, and the effects of gender and age on treatment outcomes need further study. Furthermore, SSRI usage and the presence of comorbid major depressive disorder did not significantly affect the outcomes of psychological treatment. This observation aligns with Greenberg, Phillips, Steketee, Hoeppner, and Wilhelm (2019) findings and supports the notion that psychological treatments for BDD can be helpful even for those with severe depressive symptoms (Veale et al., 2014). One potential explanation for this could be that our included studies for moderation analysis featured a relatively low overall prevalence of SSRI usage or comorbid major depressive disorder, making any potential moderating effects not statistically significant. Lastly, the session duration played a notable role in determining the efficacy of treatments. Although the overall duration of psychotherapy did not have a significant moderating effect, it is worth noting that the average duration of psychological treatment was only 12–13 weeks, whereas a longer treatment (e.g. 24 weeks; Wilhelm et al., 2019) is often recommended for BDD. One study found that many non-responders to CBT at week 12 do respond after 24 weeks of treatment (Greenberg et al., 2022). Although studies with very few sessions like CBM interventions have shown promise in experimental settings, their effectiveness in regular clinical practice remains to be fully validated. To establish the clinical utility and generalizability of CBM interventions, it is essential for future studies to focus on large-scale clinical trials and multi-center research.

### Study strengths

This study has several strengths. First, it has the largest sample size to date. Additionally, our study offers several novel insights. We found no significant differences between face-to-face and digital interventions, a finding that is particularly relevant given the increasing development and scalability of digital interventions for BDD. Secondly, the extensive moderator analysis provided new information about factors influencing treatment efficacy, such as gender differences, age-related changes in symptom response, and the impact of session duration. Notably, our analysis revealed that the presence of ERP in the treatment did not significantly impact the outcomes, and longer session duration was associated with larger effect sizes for BDD symptoms and depression. In addition, unlike previous meta-analyses that focused primarily on CBT, our study encompasses a broader range of psychological treatments, thereby providing a more holistic view of current therapeutic options. This will provide a reference for future research and clinical practice in the psychological treatments of patients with BDD. Methodologically, we conducted sensitivity analyses to ensure the robustness of the meta-analysis results, with TSA helping to mitigate random errors and assess the need for further RCTs to evaluate the effectiveness of psychological intervention for patients with BDD by calculating the RIS.

### Limitations and future lines of research

Several limitations of this study must be acknowledged. First, our analysis was limited to papers written in English, which may have excluded relevant studies published in other languages. Additionally, the sample size, as indicated by TSA, remains limited. This suggests that the effects of psychological treatments on certain outcome variables – such as BDD-related insight, quality of life, level of functioning, anxiety, and longer-term effects on depression – require further validation through additional research.

One of the most significant challenges in this meta-analysis is the considerable heterogeneity observed in the results. This heterogeneity may stem from several factors. First, the inclusion of diverse psychological treatments in the comparisons could contribute to variations in effect sizes. Second, differences in sample characteristics, such as age, gender, and severity of the condition, may affect the consistency of results. Third, variations in research quality across studies could also play a significant role in this heterogeneity. Understanding these sources of heterogeneity is crucial for interpreting our findings, and we recommend that future studies address these factors to enhance the consistency and reliability of conclusions. Furthermore, it is important to note that to comprehensively include existing studies on psychological treatments for BDD and avoid publication bias, we did not restrict our analysis to peer-reviewed papers. However, we conducted a rigorous quality assessment to minimize potential biases.

Another limitation is the exclusion of influential outlier studies during moderation analysis, which might have led to the omission of some significant moderating factors (Viechtbauer & Cheung, 2010). Additionally, many studies did not report the race and ethnicity of participants, making it unclear to whom the results apply. This highlights the need for future research to include more comprehensive demographic information to improve the generalizability of findings. Moreover, the lower proportion of males in most study samples may have limited the statistical power to detect effect sizes for this group. Future research should aim for a more balanced gender distribution to more accurately assess gender-specific effects in psychotherapeutic interventions for BDD.

Lastly, future research should explore the mechanisms by which early intervention impacts BDD-related symptoms and determine the optimal timing and strategies for such interventions. It is also imperative to conduct further RCTs focusing on digital psychological interventions for BDD to validate their potential benefits.

## Conclusion

In summary, our study provides compelling evidence that the psychological treatments evaluated effectively reduce BDD symptoms, with effects lasting up to 6 months. However, further research is necessary to reliably establish the impact of psychological treatments on other outcomes, ensuring that our conclusions are grounded in robust evidence. Our findings reveal that face-to-face and digital interventions yield similar treatment outcomes, a crucial insight given the growing focus on digital BDD interventions and their potential for scalable treatment. Additionally, patient characteristics like age, gender, and longer session durations significantly influence treatment efficacy. These novel insights emphasize the need to consider these factors in future research and clinical practice.

## Supporting information

Liu et al. supplementary material Liu et al. supplementary material

## Supplementary material

The supplementary material for this article can be found at https://doi.org/10.1017/S0033291724002733.
