# Supplementary material for: The efficacy of psychological treatments on body dysmorphic disorder: a meta-analysis and trial sequential analysis of randomized controlled trials
Source: Psychol Med. 2024 Dec 3;54(15):4048–61. doi: 10.1017/S0033291724002733 (PMC11650169; doi:10.1017/S0033291724002733)

The efficacy of psychological treatments on body dysmorphic disorder: A meta-analysis and trial sequential analysis of randomized controlled trials

Online Supplementary Material

Appendix A. PRISMA checklist

Appendix B. Search Strategy

Appendix C. Risk of Bias

Appendix D. Forest plot

Appendix E. Outlier and influence analysis

Appendix F. Funnel plot

Appendix G. Trial sequential analysis

Appendix A. PRISMA checklist

Section and Topic	Item #	Checklist item	Location where item is reported
**TITLE**			
Title	1	Identify the report as a systematic review.	p1
**ABSTRACT**			
Abstract	2	See the PRISMA 2020 for Abstracts checklist.	p2-p3
**INTRODUCTION**			
Rationale	3	Describe the rationale for the review in the context of existing knowledge.	P3-p7
Objectives	4	Provide an explicit statement of the objective(s) or question(s) the review addresses.	p7
**METHODS**			
Eligibility criteria	5	Specify the inclusion and exclusion criteria for the review and how studies were grouped for the syntheses.	p8
Information sources	6	Specify all databases, registers, websites, organisations, reference lists and other sources searched or consulted to identify studies. Specify the date when each source was last searched or consulted.	p7
Search strategy	7	Present the full search strategies for all databases, registers and websites, including any filters and limits used.	p7 and appendix B
Selection process	8	Specify the methods used to decide whether a study met the inclusion criteria of the review, including how many reviewers screened each record and each report retrieved, whether they worked independently, and if applicable, details of automation tools used in the process.	p7-p8
Data collection process	9	Specify the methods used to collect data from reports, including how many reviewers collected data from each report, whether they worked independently, any processes for obtaining or confirming data from study investigators, and if applicable, details of automation tools used in the process.	p8-p9
Data items	10a	List and define all outcomes for which data were sought. Specify whether all results that were compatible with each outcome domain in each study	p9

Section and Topic	Item #	Checklist item	Location where item is reported
		were sought (e.g. for all measures, time points, analyses), and if not, the methods used to decide which results to collect.	
	10b	List and define all other variables for which data were sought (e.g. participant and intervention characteristics, funding sources). Describe any assumptions made about any missing or unclear information.	p8
Study risk of bias assessment	11	Specify the methods used to assess risk of bias in the included studies, including details of the tool(s) used, how many reviewers assessed each study and whether they worked independently, and if applicable, details of automation tools used in the process.	p9
Effect measures	12	Specify for each outcome the effect measure(s) (e.g. risk ratio, mean difference) used in the synthesis or presentation of results.	p9-p10
Synthesis methods	13a	Describe the processes used to decide which studies were eligible for each synthesis (e.g. tabulating the study intervention characteristics and comparing against the planned groups for each synthesis (item #5)).	p8
	13b	Describe any methods required to prepare the data for presentation or synthesis, such as handling of missing summary statistics, or data conversions.	p9-p10
	13c	Describe any methods used to tabulate or visually display results of individual studies and syntheses.	p9-p10
	13d	Describe any methods used to synthesize results and provide a rationale for the choice(s). If meta-analysis was performed, describe the model(s), method(s) to identify the presence and extent of statistical heterogeneity, and software package(s) used.	p9-p10
	13e	Describe any methods used to explore possible causes of heterogeneity among study results (e.g. subgroup analysis, meta-regression).	P10-p11
	13f	Describe any sensitivity analyses conducted to assess robustness of the synthesized results.	p10
Reporting bias assessment	14	Describe any methods used to assess risk of bias due to missing results in a synthesis (arising from reporting biases).	p11
Certainty assessment	15	Describe any methods used to assess certainty (or confidence) in the body of evidence for an outcome.	p11
**RESULTS**			

Section and Topic	Item #	Checklist item	Location where item is reported
Study selection	16a	Describe the results of the search and selection process, from the number of records identified in the search to the number of studies included in the review, ideally using a flow diagram.	Figure 1
	16b	Cite studies that might appear to meet the inclusion criteria, but which were excluded, and explain why they were excluded.	NOT AVAILABEL
Study characteristics	17	Cite each included study and present its characteristics.	Table 1
Risk of bias in studies	18	Present assessments of risk of bias for each included study.	Appendix C
Results of individual studies	19	For all outcomes, present, for each study: (a) summary statistics for each group (where appropriate) and (b) an effect estimate and its precision (e.g. confidence/credible interval), ideally using structured tables or plots.	Appendix D
Results of syntheses	20a	For each synthesis, briefly summarise the characteristics and risk of bias among contributing studies.	p13 and Appendix C
	20b	Present results of all statistical syntheses conducted. If meta-analysis was done, present for each the summary estimate and its precision (e.g. confidence/credible interval) and measures of statistical heterogeneity. If comparing groups, describe the direction of the effect.	p13-p14 and table 2
	20c	Present results of all investigations of possible causes of heterogeneity among study results.	p14 and table 3
	20d	Present results of all sensitivity analyses conducted to assess the robustness of the synthesized results.	p14 and appendix E
Reporting biases	21	Present assessments of risk of bias due to missing results (arising from reporting biases) for each synthesis assessed.	p15 and Appendix C

Section and Topic	Item #	Checklist item	Location where item is reported
Certainty of evidence	22	Present assessments of certainty (or confidence) in the body of evidence for each outcome assessed.	p16 and appendix G
**DISCUSSION**			
Discussion	23a	Provide a general interpretation of the results in the context of other evidence.	p17-p19
	23b	Discuss any limitations of the evidence included in the review.	P20
	23c	Discuss any limitations of the review processes used.	P20
	23d	Discuss implications of the results for practice, policy, and future research.	P18-19
**OTHER INFORMATION**			
Registration and protocol	24a	Provide registration information for the review, including register name and registration number, or state that the review was not registered.	p7
	24b	Indicate where the review protocol can be accessed, or state that a protocol was not prepared.	p7
	24c	Describe and explain any amendments to information provided at registration or in the protocol.	p7
Support	25	Describe sources of financial or non-financial support for the review, and the role of the funders or sponsors in the review.	P1
Competing interests	26	Declare any competing interests of review authors.	P1
Availability of data, code and other materials	27	Report which of the following are publicly available and where they can be found: template data collection forms; data extracted from included studies; data used for all analyses; analytic code; any other materials used in the review.	Appendix

From: Page MJ, McKenzie JE, Bossuyt PM, Boutron I, Hoffmann TC, Mulrow CD, et al. The PRISMA 2020 statement: an updated guideline for reporting systematic reviews. BMJ 2021;372:n71. doi: 10.1136/bmj.n71For more information, visit: http://www.prisma-statement.org/

Appendix B. Search Strategy

Web of Science

TS= (("muscle dysmorphia" OR "dysmorphophobia" OR "dysmorphia" OR "body dysmorphic disorder") AND ("Randomized Controlled Trial" OR Controlled Clinical Trial OR random allocation OR "randomised" OR randomly OR "controlled clinical trial" OR random* OR "clinical trial"))

Embase

#1	'muscle dysmorphia'/exp OR 'dysmorphophobia':ab,ti OR 'dysmorphia':ab,ti OR 'body dysmorphic disorder*':ab,ti
#2	#1 AND ('controlled clinical trial'/de OR 'randomized controlled trial'/de)

PsycINFO

SI	AB "muscle dysmorphia" OR AB "dysmorphophobia" OR AB dysmorphia OR AB "body dysmorphic disorder"
S2	AB "Randomized Controlled Trial" OR AB "Controlled Clinical Trial" OR AB "random allocation" OR AB randomised OR AB randomly OR AB "RCT" OR AB "controlled clinical trial" OR AB random* OR AB "clinical trial"
S3	S1 AND S2

Cochrane trail

#1	MeSH descriptor: [Body Dysmorphic Disorder] explode all trees
#2	("muscle dysmorphia"):ti,ab,kw OR ("dysmorphophobia"):ti,ab,kw OR (dysmorphia):ti,ab,kw OR ("body dysmorphic disorder"):ti,ab,kw

PubMed

#1	(("muscle dysmorphia"[Title/Abstract]) OR ("dysmorphophobia"[Title/Abstract]) OR ("dysmorphia"[Title/Abstract]) OR ("body dysmorphic disorder*"[Title/Abstract]))
#2	Filters applied: Clinical Trial, Randomized Controlled Trial.

Scopus

((TITLE-ABS-KEY ("muscle dysmorphia") OR TITLE-ABS-KEY ("dysmorphophobia") OR TITLE-ABS-KEY ("dysmorphia") OR TITLE-ABS-KEY ("body dysmorphic disorder"))) AND ((TITLE-ABS-KEY ("randomized controlled trial") OR TITLE-ABS-KEY ("controlled trial") OR TITLE-ABS-KEY (randomized) OR TITLE-ABS-KEY (trial*) OR TITLE-ABS-KEY (random*) OR TITLE-ABS-KEY ("RCT"))

Appendix C. Risk of Bias

Study ID	D1	D2	D3	D4	D5	Overall
Enander et al. (2016)	+	+	+	+	+	+
Guan & Zhu (2023)	+	+	+	+	+	+
Mataix-Cols et al. (2015)	+	+	+	+	+	+
Mohajerin et al. (2019)	+	+	+	+	+	+
Rabiei et al. (2012)	!	+	+	!	+	!
Ritter et al. (2023)	+	+	!	+	+	!
Rosen et al. (1995)	!	+	+	!	+	!
Summers and Cougle (2016)	!	+	+	-	+	-
Torkian et al. (2022)	+	+	+	!	+	!
Veale et al. (2014)	+	+	!	+	+	+
Veale et al. (1996)	!	+	+	!	+	!
Wilhelm et al. (2019)	+	+	!	+	+	!
Wilhelm et al. (2014)	+	+	!	+	+	!
Wilhelm et al. (2022)	+	+	+	+	+	+
Wilver and Cougle (2019)	+	+	+	+	+	+

+ Low risk

! Some concerns

- High risk

Note: D1. Bias arising from the randomization process; D2: Bias due to deviations from intended intervention; D3: Bias due to missing outcome data; D4: Bias in measurement of the outcome; D5: Bias in selection of the reported result.

Appendix D. Forest plot

Figure D1. Forest plot for BDD symptoms at post treatment

A Effect of psychological intervention for BDD symptoms

Study name	Hedges's g	Standard error	Variance	Lower limit	Upper limit	Z-Value	p-Value
Enander et al. (2016)	-0.947	0.217	0.047	-1.372	-0.521	-4.361	0.000
Guan & Zhu (2023)	-0.883	0.199	0.040	-1.274	-0.493	-4.430	0.000
Mataix-Cols et al. (2015)	-1.103	0.389	0.151	-1.865	-0.340	-2.833	0.005
Mohajerin et al. (2019)	-9.110	0.596	0.355	-10.278	-7.942	-15.288	0.000
Rabiei et al. (2012)	-1.364	0.480	0.230	-2.303	-0.424	-2.844	0.004
Ritter et al. (2023)	-0.778	0.376	0.142	-1.515	-0.040	-2.067	0.039
Rosen et al. (1995)	-2.261	0.345	0.119	-2.937	-1.584	-6.546	0.000
Summers and Cougle (2016)	-0.845	0.332	0.110	-1.496	-0.194	-2.545	0.011
Torkian et al. (2022)	-0.730	0.346	0.120	-1.409	-0.051	-2.109	0.035
Veale et al. (2014)	-0.971	0.333	0.111	-1.623	-0.320	-2.921	0.003
Veale et al. (1996)	-1.812	0.528	0.279	-2.848	-0.777	-3.431	0.001
Wilhelm et al. (2019)	-0.549	0.211	0.044	-0.963	-0.136	-2.604	0.009
Wilhelm et al. (2014)	-0.426	0.365	0.134	-1.142	0.290	-1.165	0.244
Wilhelm et al. (2022)	-1.435	0.270	0.073	-1.965	-0.905	-5.307	0.000
Wilver and Cougle (2019)	-0.041	0.281	0.079	-0.592	0.511	-0.144	0.885
	-1.473	0.323	0.104	-2.105	-0.841	-4.568	0.000

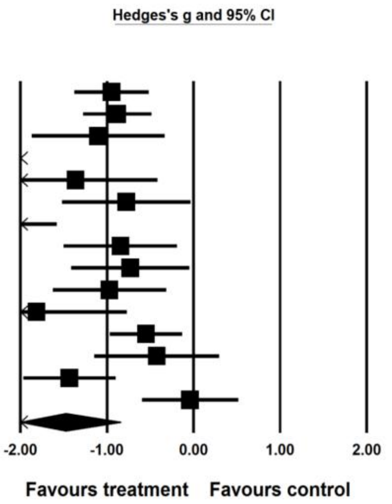

B Effect of psychological intervention for BDD symptoms

Study name	Hedges's g	Standard error	Variance	Lower limit	Upper limit	Z-Value	p-Value
Enander et al. (2016)	-0.947	0.217	0.047	-1.372	-0.521	-4.361	0.000
Guan & Zhu (2023)	-0.883	0.199	0.040	-1.274	-0.493	-4.430	0.000
Mataix-Cols et al. (2015)	-1.103	0.389	0.151	-1.865	-0.340	-2.833	0.005
Rabiei et al. (2012)	-1.364	0.480	0.230	-2.303	-0.424	-2.844	0.004
Ritter et al. (2023)	-0.778	0.376	0.142	-1.515	-0.040	-2.067	0.039
Rosen et al. (1995)	-2.261	0.345	0.119	-2.937	-1.584	-6.546	0.000
Summers and Cougle (2016)	-0.845	0.332	0.110	-1.496	-0.194	-2.545	0.011
Torkian et al. (2022)	-0.730	0.346	0.120	-1.409	-0.051	-2.109	0.035
Veale et al. (2014)	-0.971	0.333	0.111	-1.623	-0.320	-2.921	0.003
Veale et al. (1996)	-1.812	0.528	0.279	-2.848	-0.777	-3.431	0.001
Wilhelm et al. (2019)	-0.549	0.211	0.044	-0.963	-0.136	-2.604	0.009
Wilhelm et al. (2014)	-0.426	0.365	0.134	-1.142	0.290	-1.165	0.244
Wilhelm et al. (2022)	-1.435	0.270	0.073	-1.965	-0.905	-5.307	0.000
Wilver and Cougle (2019)	-0.041	0.281	0.079	-0.592	0.511	-0.144	0.885
	-0.966	0.141	0.020	-1.243	-0.688	-6.828	0.000

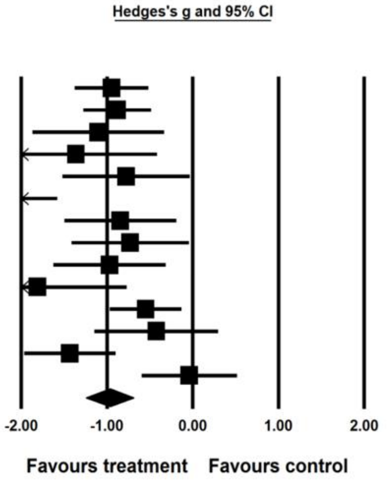

Note: A) with outliers; B) exclude outliers.

Figure D2. Forest plot for BDD symptoms at follow-up

A Effect of psychological intervention for BDD symptoms

Study name	Statistics for each study							Hedges's g and 95% CI
	Hedges's g	Standard error	Variance	Lower limit	Upper limit	Z-Value	p-Value	
Enander et al. (2016)	-0.859	0.224	0.050	-1.297	-0.420	-3.838	0.000	
Guan & Zhu (2023)	-0.856	0.210	0.044	-1.267	-0.446	-4.086	0.000	
Mataix-Cols et al. (2015)	-0.897	0.395	0.156	-1.671	-0.123	-2.272	0.023	
Mohajerin et al. (2019)	-8.666	0.569	0.324	-9.783	-7.550	-15.219	0.000	
Rabiei et al. (2012)	-1.313	0.476	0.227	-2.245	-0.380	-2.758	0.006	
Summers and Cougle (2016)	-0.813	0.377	0.142	-1.551	-0.075	-2.158	0.031	
Veale et al. (2014)	-1.098	0.351	0.123	-1.786	-0.410	-3.127	0.002	
Wilhelm et al. (2019)	-0.419	0.230	0.053	-0.869	0.031	-1.824	0.068	
Wilver and Cougle (2019)	-0.049	0.296	0.088	-0.630	0.532	-0.165	0.869	
	-1.592	0.510	0.260	-2.592	-0.592	-3.120	0.002	

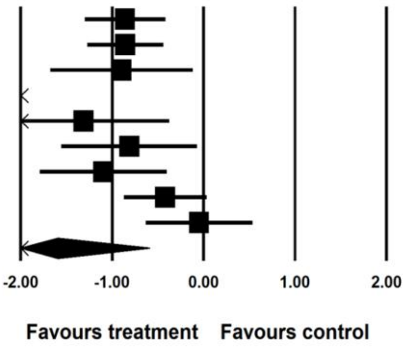

B Effect of psychological intervention for BDD symptoms

Study name	Statistics for each study							Hedges's g and 95% CI
	Hedges's g	Standard error	Variance	Lower limit	Upper limit	Z-Value	p-Value	
Enander et al. (2016)	-0.859	0.224	0.050	-1.297	-0.420	-3.838	0.000	
Guan & Zhu (2023)	-0.856	0.210	0.044	-1.267	-0.446	-4.086	0.000	
Mataix-Cols et al. (2015)	-0.897	0.395	0.156	-1.671	-0.123	-2.272	0.023	
Rabiei et al. (2012)	-1.313	0.476	0.227	-2.245	-0.380	-2.758	0.006	
Summers and Cougle (2016)	-0.813	0.377	0.142	-1.551	-0.075	-2.158	0.031	
Veale et al. (2014)	-1.098	0.351	0.123	-1.786	-0.410	-3.127	0.002	
Wilhelm et al. (2019)	-0.419	0.230	0.053	-0.869	0.031	-1.824	0.068	
Wilver and Cougle (2019)	-0.049	0.296	0.088	-0.630	0.532	-0.165	0.869	
	-0.731	0.129	0.017	-0.984	-0.478	-5.671	0.000	

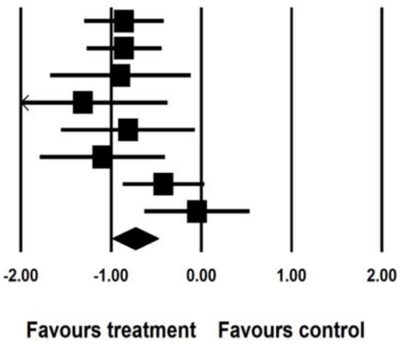

Note: A) with outliers; B) exclude outliers.

Figure D3. Forest plot for depression symptoms at post treatment

A Effect of psychological intervention for depression symptoms

Study name	Hedges's g	Standard error	Variance	Lower limit	Upper limit	Z-Value	p-Value
Enander et al. (2016)	-0.429	0.208	0.043	-0.837	-0.021	-2.063	0.039
Guan & Zhu (2023)	-0.766	0.197	0.039	-1.153	-0.380	-3.886	0.000
Mataix-Cols et al. (2015)	-0.911	0.394	0.155	-1.683	-0.140	-2.316	0.021
Mohajerin et al. (2019)	-9.678	0.630	0.397	-10.913	-8.444	-15.365	0.000
Ritter et al. (2023)	-0.507	0.368	0.136	-1.229	0.215	-1.376	0.169
Summers and Cougle (2016)	-0.283	0.319	0.102	-0.908	0.343	-0.885	0.376
Torkian et al. (2022)	-0.703	0.346	0.119	-1.381	-0.026	-2.035	0.042
Veale et al. (2014)	-0.574	0.320	0.103	-1.202	0.054	-1.791	0.073
Veale et al. (1996)	-1.656	0.515	0.265	-2.665	-0.647	-3.218	0.001
Wilhelm et al. (2019)	-0.394	0.209	0.044	-0.803	0.016	-1.884	0.060
Wilhelm et al. (2014)	0.126	0.362	0.131	-0.583	0.835	0.348	0.728
Wilhelm et al. (2022)	-0.730	0.257	0.066	-1.233	-0.226	-2.837	0.005
Wilver and Cougle (2019)	0.076	0.281	0.079	-0.475	0.628	0.272	0.786
	-1.157	0.358	0.128	-1.858	-0.456	-3.234	0.001

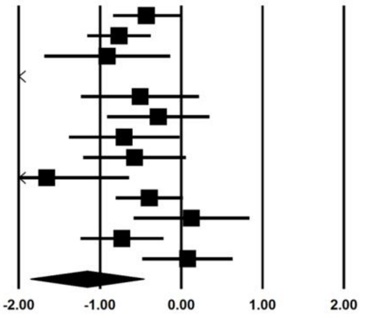

B Effect of psychological intervention for depression symptoms

Study name	Hedges's g	Standard error	Variance	Lower limit	Upper limit	Z-Value	p-Value
Enander et al. (2016)	-0.429	0.208	0.043	-0.837	-0.021	-2.063	0.039
Guan & Zhu (2023)	-0.766	0.197	0.039	-1.153	-0.380	-3.886	0.000
Mataix-Cols et al. (2015)	-0.911	0.394	0.155	-1.683	-0.140	-2.316	0.021
Ritter et al. (2023)	-0.507	0.368	0.136	-1.229	0.215	-1.376	0.169
Summers and Cougle (2016)	-0.283	0.319	0.102	-0.908	0.343	-0.885	0.376
Torkian et al. (2022)	-0.703	0.346	0.119	-1.381	-0.026	-2.035	0.042
Veale et al. (2014)	-0.574	0.320	0.103	-1.202	0.054	-1.791	0.073
Veale et al. (1996)	-1.656	0.515	0.265	-2.665	-0.647	-3.218	0.001
Wilhelm et al. (2019)	-0.394	0.209	0.044	-0.803	0.016	-1.884	0.060
Wilhelm et al. (2014)	0.126	0.362	0.131	-0.583	0.835	0.348	0.728
Wilhelm et al. (2022)	-0.730	0.257	0.066	-1.233	-0.226	-2.837	0.005
Wilver and Cougle (2019)	0.076	0.281	0.079	-0.475	0.628	0.272	0.786
	-0.512	0.105	0.011	-0.719	-0.306	-4.871	0.000

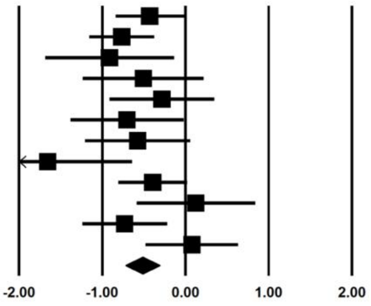

Note: A) with outliers; B) exclude outliers.

Figure D4. Forest plot for depression symptoms at follow-up

A Effect of psychological therapy on symptoms of depression

Statistics for each study

Study name	Hedges's g	Standard error	Variance	Lower limit	Upper limit	Z-Value	p-Value
Enander et al. (2016)	-0.578	0.220	0.048	-1.008	-0.148	-2.632	0.008
Guan & Zhu (2023)	-0.842	0.209	0.044	-1.253	-0.432	-4.024	0.000
Mataix-Cols et al. (2015)	-0.236	0.389	0.151	-0.997	0.526	-0.607	0.544
Mohajerin et al. (2019)	-8.091	0.535	0.287	-9.140	-7.042	-15.113	0.000
Summers and Cougle (2016)	-0.051	0.361	0.131	-0.759	0.657	-0.140	0.888
Veale et al. (2014)	-0.908	0.344	0.118	-1.581	-0.234	-2.642	0.008
Wilhelm et al. (2019)	-0.264	0.217	0.047	-0.690	0.161	-1.217	0.223
Wilver and Cougle (2019)	0.165	0.297	0.088	-0.417	0.747	0.554	0.579
	-1.289	0.556	0.309	-2.378	-0.199	-2.319	0.020

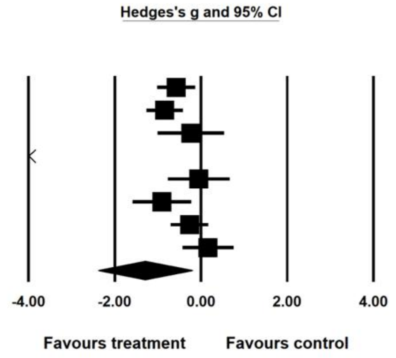

B Effect of psychological intervention for depression symptoms

Statistics for each study

Study name	Hedges's g	Standard error	Variance	Lower limit	Upper limit	Z-Value	p-Value
Enander et al. (2016)	-0.578	0.220	0.048	-1.008	-0.148	-2.632	0.008
Guan & Zhu (2023)	-0.842	0.209	0.044	-1.253	-0.432	-4.024	0.000
Mataix-Cols et al. (2015)	-0.236	0.389	0.151	-0.997	0.526	-0.607	0.544
Summers and Cougle (2016)	-0.051	0.361	0.131	-0.759	0.657	-0.140	0.888
Veale et al. (2014)	-0.908	0.344	0.118	-1.581	-0.234	-2.642	0.008
Wilhelm et al. (2019)	-0.107	0.248	0.061	-0.592	0.378	-0.432	0.666
Wilver and Cougle (2019)	0.165	0.297	0.088	-0.417	0.747	0.554	0.579
	-0.391	0.159	0.025	-0.703	-0.079	-2.454	0.014

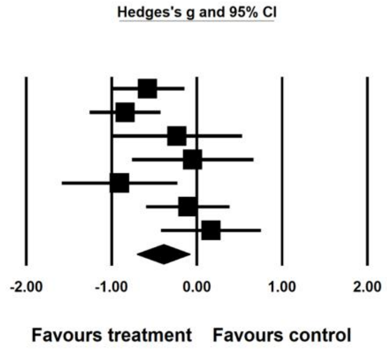

Note: A) with outliers; B) exclude outliers.

Figure D5. Forest plot for Insight at post treatment

A Effect of psychological intervention for Insight

Study name	Hedges's g	Standard error	Variance	Lower limit	Upper limit	Z-Value	p-Value
Guan & Zhu (2023)	-0.627	0.195	0.038	-1.009	-0.245	-3.215	0.001
Mataix-Cols et al. (2015)	-1.238	0.403	0.162	-2.027	-0.449	-3.075	0.002
Mohajerin et al. (2019)	-12.204	0.783	0.613	-13.738	-10.670	-15.592	0.000
Ritter et al. (2023)	-1.063	0.388	0.150	-1.823	-0.303	-2.742	0.006
Veale et al. (2014)	-0.738	0.325	0.105	-1.375	-0.102	-2.273	0.023
Wilhelm et al. (2019)	0.056	0.207	0.043	-0.350	0.462	0.270	0.787
Wilhelm et al. (2014)	-0.355	0.364	0.133	-1.068	0.359	-0.973	0.330
Wilhelm et al. (2022)	-0.939	0.256	0.066	-1.441	-0.438	-3.669	0.000
Wilver and Cougle (2019)	0.000	0.281	0.079	-0.551	0.551	0.000	1.000
	-1.705	0.545	0.297	-2.772	-0.638	-3.131	0.002

Hedges's g and 95% CI

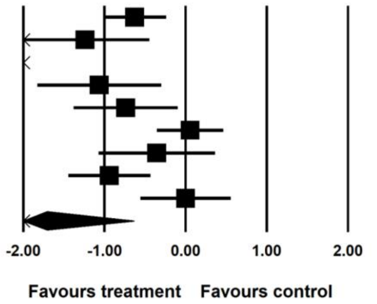

B Effect of psychological intervention for Insight

Study name	Hedges's g	Standard error	Variance	Lower limit	Upper limit	Z-Value	p-Value
Guan & Zhu (2023)	-0.627	0.195	0.038	-1.009	-0.245	-3.215	0.001
Mataix-Cols et al. (2015)	-1.238	0.403	0.162	-2.027	-0.449	-3.075	0.002
Ritter et al. (2023)	-1.063	0.388	0.150	-1.823	-0.303	-2.742	0.006
Veale et al. (2014)	-0.738	0.325	0.105	-1.375	-0.102	-2.273	0.023
Wilhelm et al. (2019)	0.056	0.207	0.043	-0.350	0.462	0.270	0.787
Wilhelm et al. (2014)	-0.355	0.364	0.133	-1.068	0.359	-0.973	0.330
Wilhelm et al. (2022)	-0.939	0.256	0.066	-1.441	-0.438	-3.669	0.000
Wilver and Cougle (2019)	0.000	0.281	0.079	-0.551	0.551	0.000	1.000
	-0.568	0.170	0.029	-0.901	-0.234	-3.334	0.001

Hedges's g and 95% CI

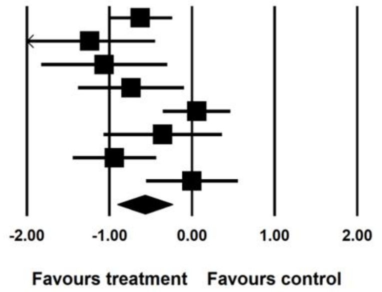

Note: A) with outliers; B) exclude outliers.

Figure D6. Forest plot for Insight at follow-up

A Effect of psychological intervention for Insight

Study name	Hedges's g	Standard error	Variance	Lower limit	Upper limit	Z-Value	p-Value
Guan & Zhu (2023)	-0.909	0.211	0.044	-1.322	-0.496	-4.312	0.000
Mataix-Cols et al. (2015)	-0.251	0.377	0.142	-0.991	0.488	-0.667	0.505
Mohajerin et al. (2019)	-13.005	0.832	0.692	-14.635	-11.375	-15.639	0.000
Veale et al. (2014)	-1.380	0.365	0.133	-2.095	-0.665	-3.784	0.000
Wilhelm et al. (2019)	-0.024	0.227	0.052	-0.470	0.421	-0.107	0.914
Wilver and Cougle (2019)	-0.284	0.298	0.089	-0.868	0.300	-0.953	0.341
	-2.431	0.868	0.753	-4.132	-0.730	-2.801	0.005

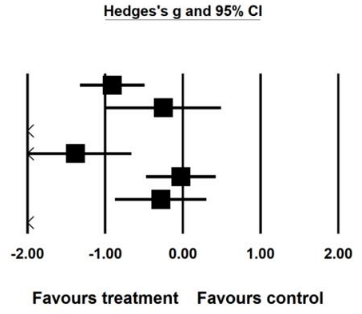

B Effect of psychological intervention for Insight

Study name	Hedges's g	Standard error	Variance	Lower limit	Upper limit	Z-Value	p-Value
Guan & Zhu (2023)	-0.909	0.211	0.044	-1.322	-0.496	-4.312	0.000
Mataix-Cols et al. (2015)	-0.251	0.377	0.142	-0.991	0.488	-0.667	0.505
Veale et al. (2014)	-1.380	0.365	0.133	-2.095	-0.665	-3.784	0.000
Wilhelm et al. (2019)	-0.024	0.227	0.052	-0.470	0.421	-0.107	0.914
Wilver and Cougle (2019)	-0.284	0.298	0.089	-0.868	0.300	-0.953	0.341
	-0.555	0.244	0.060	-1.034	-0.076	-2.272	0.023

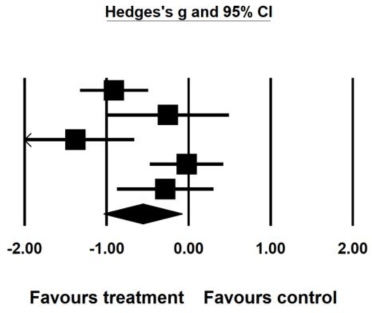

Note: A) with outliers; B) exclude outliers

Figure D7. Forest plot for quality of life at post treatment and follow-up

A Effect of psychological intervention for quality of life

Study name	Hedges's g	Standard error	Variance	Lower limit	Upper limit	Z-Value	p-Value
Enander et al. (2016)	0.229	0.208	0.043	-0.178	0.636	1.103	0.270
Mataix-Cols et al. (2015)	0.892	0.416	0.173	0.076	1.707	2.143	0.032
Ritter et al. (2023)	0.020	0.363	0.131	-0.691	0.730	0.055	0.956
Veale et al. (2014)	0.765	0.326	0.106	0.127	1.403	2.349	0.019
Wilhelm et al. (2019)	0.221	0.212	0.045	-0.196	0.637	1.038	0.299
Wilhelm et al. (2022)	0.783	0.257	0.066	0.280	1.287	3.050	0.002
	0.439	0.138	0.019	0.169	0.708	3.186	0.001

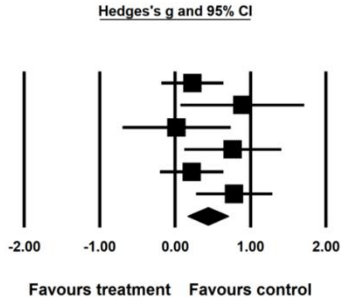

B Effect of psychological intervention for quality of life

Study name	Hedges's g	Standard error	Variance	Lower limit	Upper limit	Z-Value	p-Value
Enander et al. (2016)	0.531	0.219	0.048	0.102	0.960	2.427	0.015
Mataix-Cols et al. (2015)	0.084	0.420	0.176	-0.739	0.906	0.200	0.841
Veale et al. (2014)	0.747	0.338	0.114	0.085	1.410	2.210	0.027
Wilhelm et al. (2019)	0.030	0.244	0.060	-0.449	0.509	0.123	0.902
	0.356	0.169	0.029	0.024	0.688	2.102	0.036

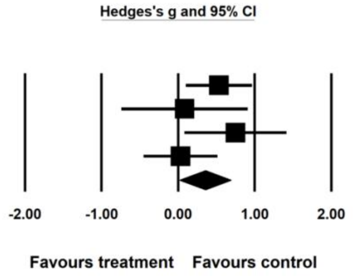

Note: A) Effect for quality of life at post treatment; B) Effect for quality of life at follow-up

Figure D8. Forest plot for Psychosocial functioning at post treatment and follow-up

A Effect of psychological intervention for psychosocial functioning

Study name	Hedges's g	Standard error	Variance	Lower limit	Upper limit	Z-Value	p-Value
Enander et al. (2016)	0.677	0.212	0.045	0.262	1.092	3.199	0.001
Mataix-Cols et al. (2015)	0.514	0.373	0.139	-0.218	1.246	1.377	0.169
Ritter et al. (2023)	0.543	0.369	0.136	-0.181	1.267	1.470	0.142
Wilhelm et al. (2019)	0.152	0.213	0.045	-0.265	0.570	0.715	0.475
Wilhelm et al. (2014)	-0.148	0.362	0.131	-0.857	0.561	-0.408	0.683
Wilhelm et al. (2022)	0.822	0.258	0.066	0.317	1.327	3.190	0.001
	0.451	0.146	0.021	0.165	0.736	3.096	0.002

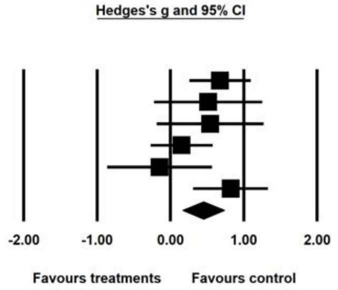

B Effect of psychological intervention for psychosocial functioning

Study name	Hedges's g	Standard error	Variance	Lower limit	Upper limit	Z-Value	p-Value
Enander et al. (2016)	0.702	0.221	0.049	0.270	1.134	3.184	0.001
Mataix-Cols et al. (2015)	0.781	0.390	0.152	0.016	1.546	2.001	0.045
Wilhelm et al. (2019)	0.382	0.229	0.053	-0.067	0.832	1.668	0.095
	0.582	0.147	0.022	0.293	0.870	3.951	0.000

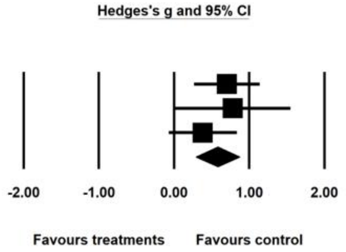

Note: A) Effect for Psychosocial functioning at post treatment; B) Effect for Psychosocial functioning at follow-up

Figure D9. Forest plot for Anxiety at post treatment

A Effect of psychological therapy on symptoms of anxiety

Study name	Hedges's g	Standard error	Variance	Lower limit	Upper limit	Z-Value	p-Value
Guan & Zhu (2023)	-0.845	0.199	0.039	-1.234	-0.456	-4.254	0.000
Summers and Cougle (2016)	-0.691	0.327	0.107	-1.333	-0.050	-2.112	0.035
Torkian et al. (2022)	-0.713	0.346	0.120	-1.391	-0.036	-2.063	0.039
Veale et al. (2014)	-0.755	0.325	0.106	-1.393	-0.118	-2.322	0.020
Veale et al. (1996)	-0.117	0.439	0.193	-0.978	0.744	-0.266	0.790
Wilver and Cougle (2019)	0.206	0.282	0.080	-0.346	0.759	0.732	0.464
	-0.510	0.187	0.035	-0.878	-0.143	-2.726	0.006

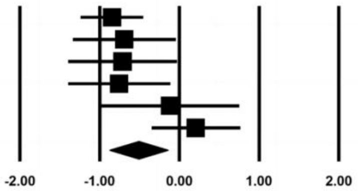

B Effect of psychological intervention for anxiety symptoms

Study name	Hedges's g	Standard error	Variance	Lower limit	Upper limit	Z-Value	p-Value
Guan & Zhu (2023)	-0.845	0.199	0.039	-1.234	-0.456	-4.254	0.000
Summers and Cougle (2016)	-0.691	0.327	0.107	-1.333	-0.050	-2.112	0.035
Torkian et al. (2022)	-0.713	0.346	0.120	-1.391	-0.036	-2.063	0.039
Veale et al. (2014)	-0.755	0.325	0.106	-1.393	-0.118	-2.322	0.020
Veale et al. (1996)	-0.117	0.439	0.193	-0.978	0.744	-0.266	0.790
	-0.721	0.132	0.017	-0.979	-0.463	-5.475	0.000

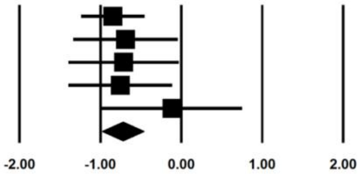

Note: A) with outliers; B) exclude outliers

Figure D10. Forest plot for Anxiety at follow-up

A Effect of psychological intervention for anxiety symptoms

Study name	Hedges's g	Standard error	Variance	Lower limit	Upper limit	Z-Value	p-Value
Guan & Zhu (2023)	-0.802	0.209	0.043	-1.211	-0.394	-3.848	0.000
Summers and Cougle (2016)	-0.158	0.362	0.131	-0.867	0.551	-0.437	0.662
Veale et al. (2014)	-0.762	0.339	0.115	-1.425	-0.098	-2.251	0.024
Wilver and Cougle (2019)	0.109	0.297	0.088	-0.473	0.690	0.367	0.714
	-0.428	0.238	0.056	-0.894	0.037	-1.803	0.071

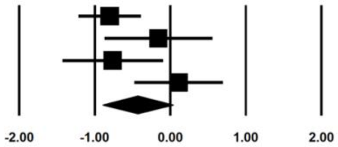

B Effect of psychological intervention for anxiety symptoms

Study name	Hedges's g	Standard error	Variance	Lower limit	Upper limit	Z-Value	p-Value
Guan & Zhu (2023)	-0.802	0.209	0.043	-1.211	-0.394	-3.848	0.000
Summers and Cougle (2016)	-0.158	0.362	0.131	-0.867	0.551	-0.437	0.662
Veale et al. (2014)	-0.762	0.339	0.115	-1.425	-0.098	-2.251	0.024
	-0.648	0.184	0.034	-1.010	-0.287	-3.513	0.000

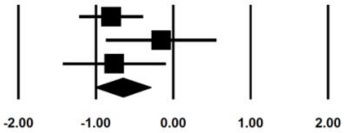

Note: A) with outliers; B) exclude outliers

Appendix E: Outlier and influence analysis

Figure E1. Outlier and influence analysis for BDD symptoms

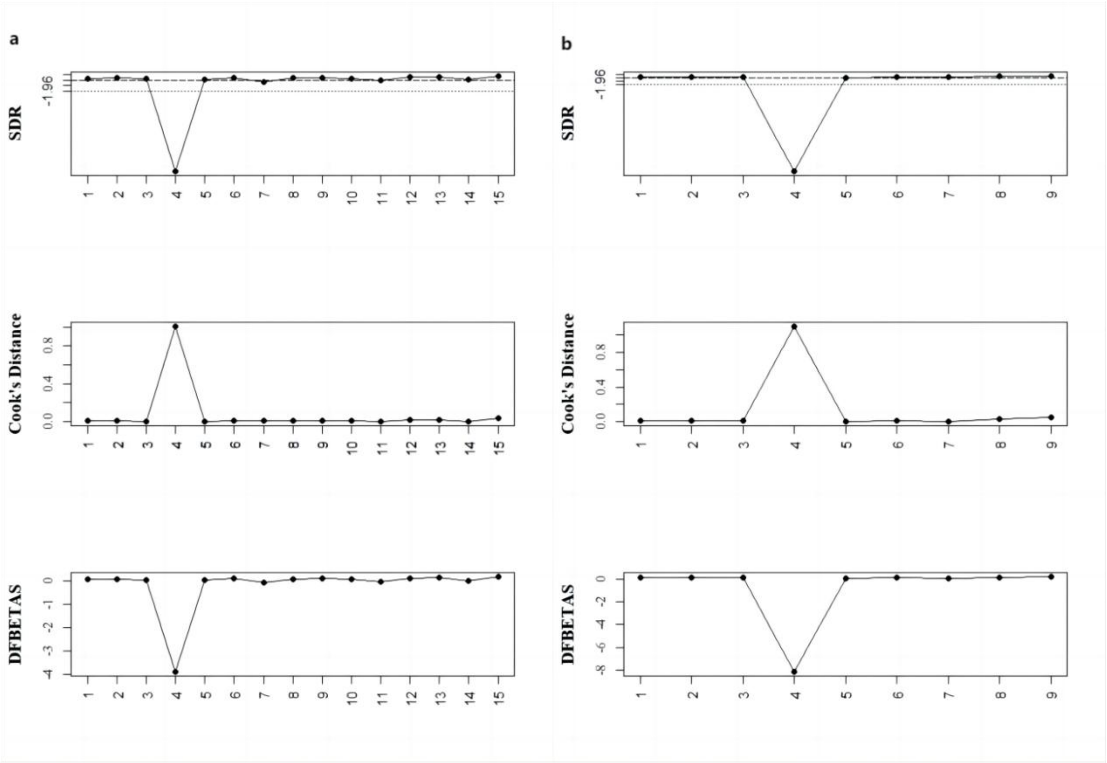

a:Outlier and influence analysis for Body dysmorphic disorder symptoms at post-treatment;b:Outlier and influence analysis for Body dysmorphic disorder symptoms at follow-up

Note. Sample 4 = Body dysmorphic disorder symptoms measured by BDD-YBOCS in Mohajerin et al. (2019).

Figure E2. Outlier and influence analysis for Depression

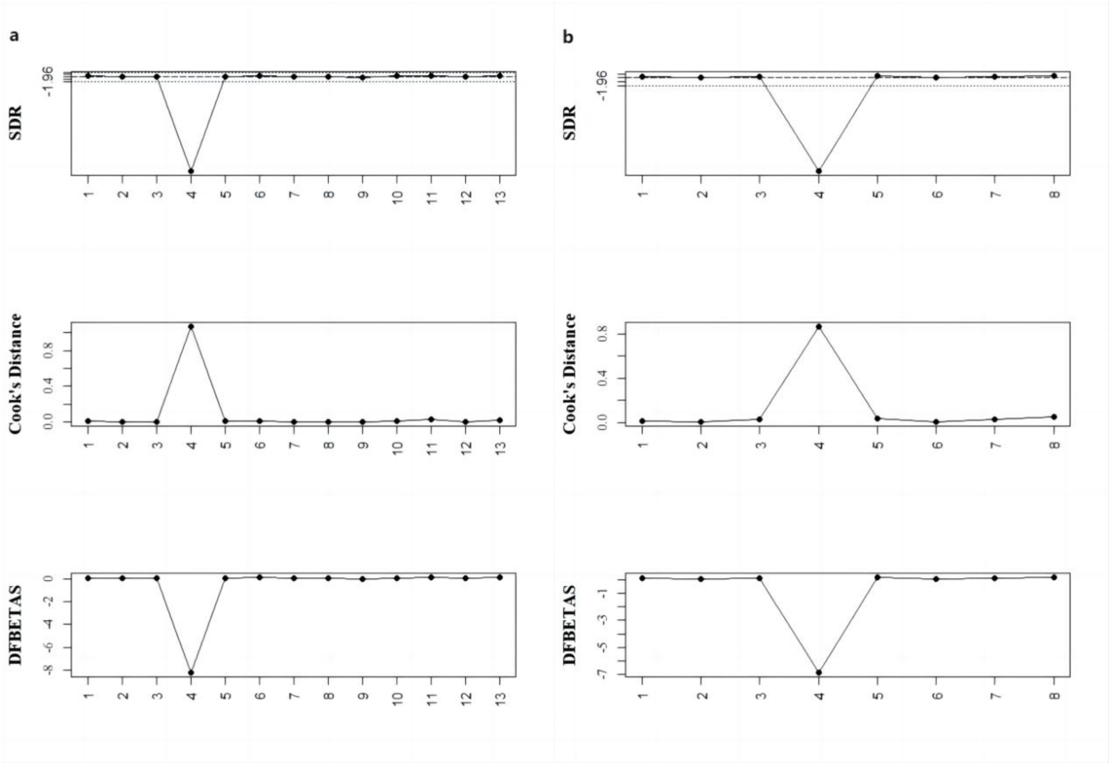

a:Outlier and influence analysis for Depression at post-treatment; b: Outlier and influence analysis for Depression at follow-up

Note. Sample 4 = Depression measured by BDI-II in Mohajerin et al. (2019).

Figure E3. Outlier and influence analysis for Insight

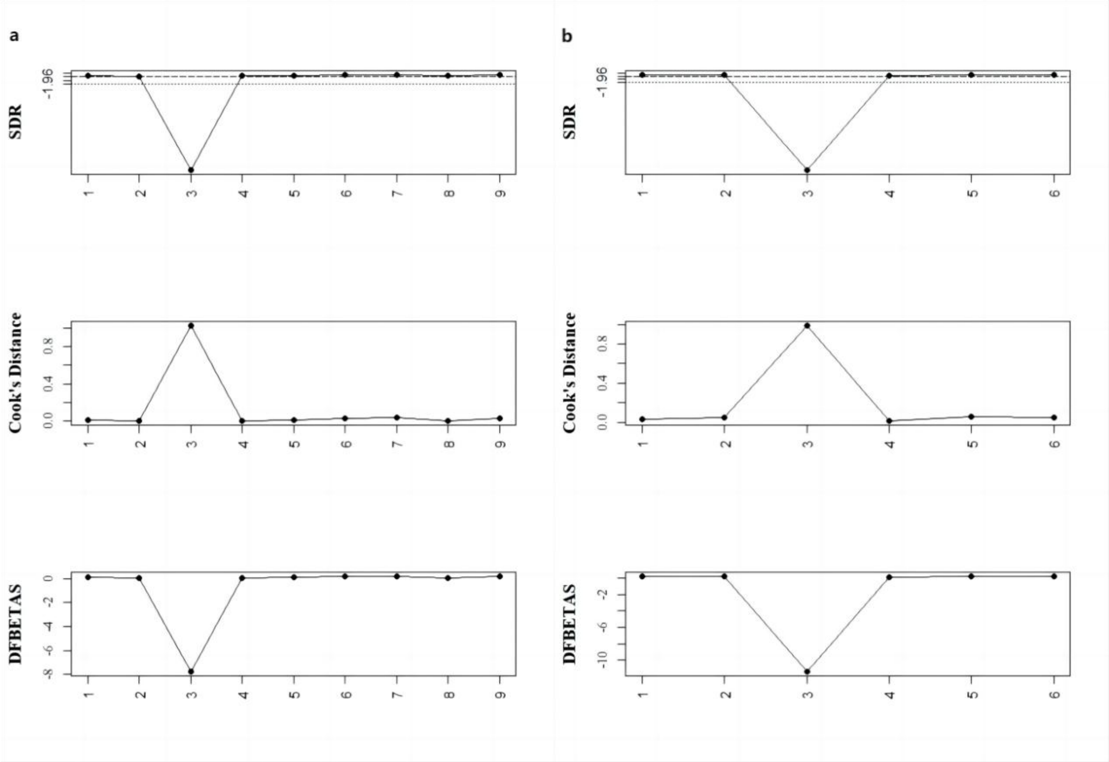

a:Outlier and influence analysis for Insight at post-treatment；b: Outlier and influence analysis for Anxiety at follow-up

Note. Sample 3 = Insight measured by BABS in Mohajerin et al. (2019).

Figure E4. Outlier and influence analysis for Anxiety

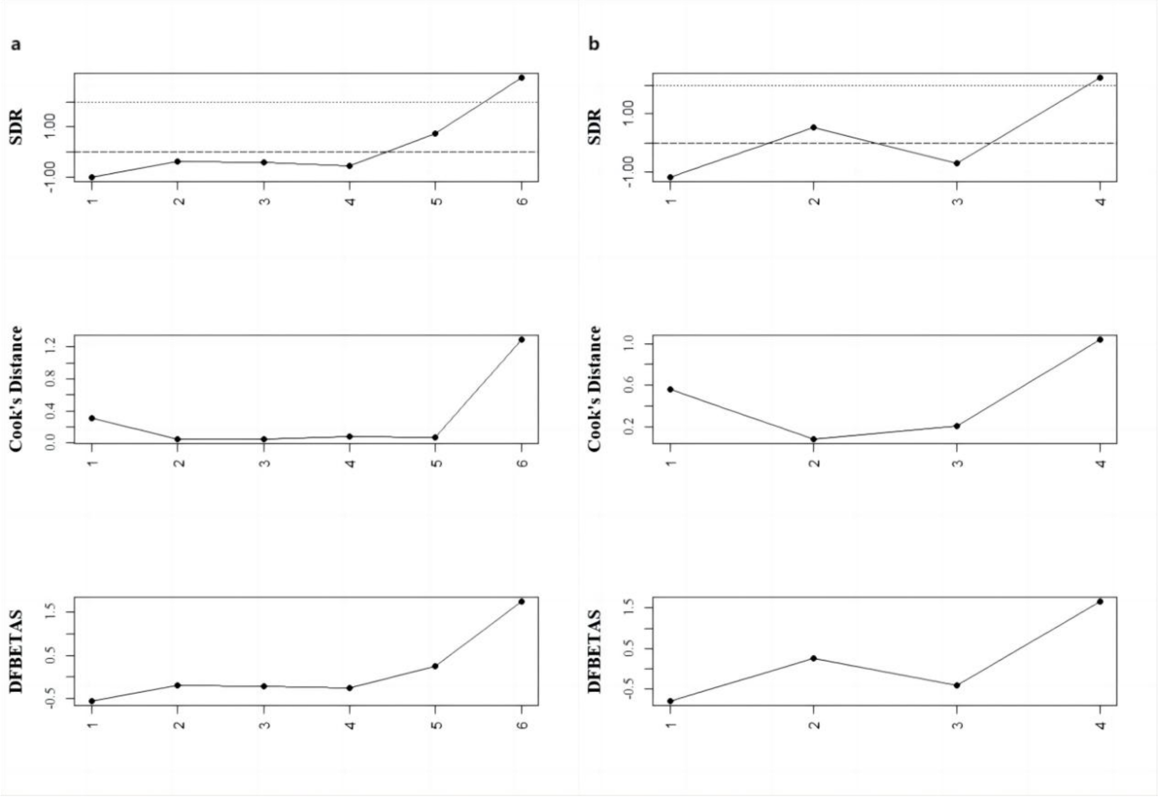

a:Outlier and influence analysis for Anxiety at post-treatment

Note. Sample 6 = Anxiety measured by BAI in Wilver & Cougle (2019).

b: Outlier and influence analysis for Anxiety at follow-up

Note. Sample 4 = Anxiety measured by BAI in Wilver & Cougle (2019).

Figure E5. Outlier and influence analysis for Psychosocial functioning

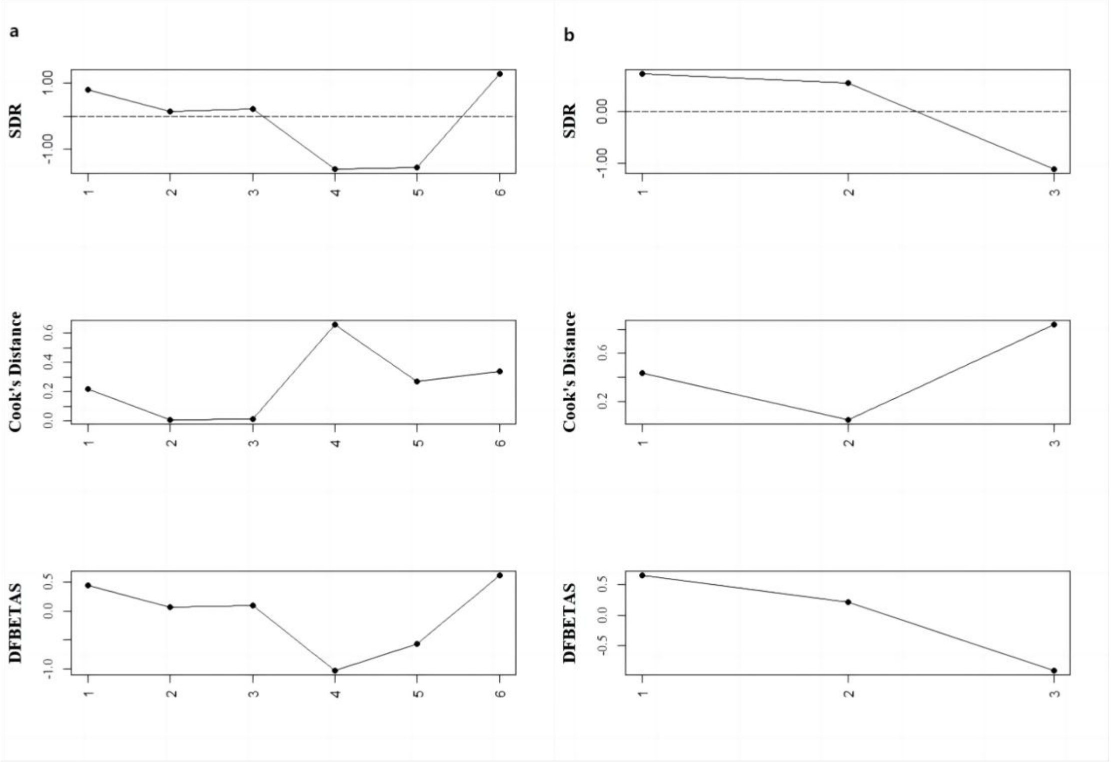

a:Outlier and influence analysis for Functional impairment at post-treatment

b: Outlier and influence analysis for Functional impairment at follow-up

Figure E6. Outlier and influence analysis for Quality of life

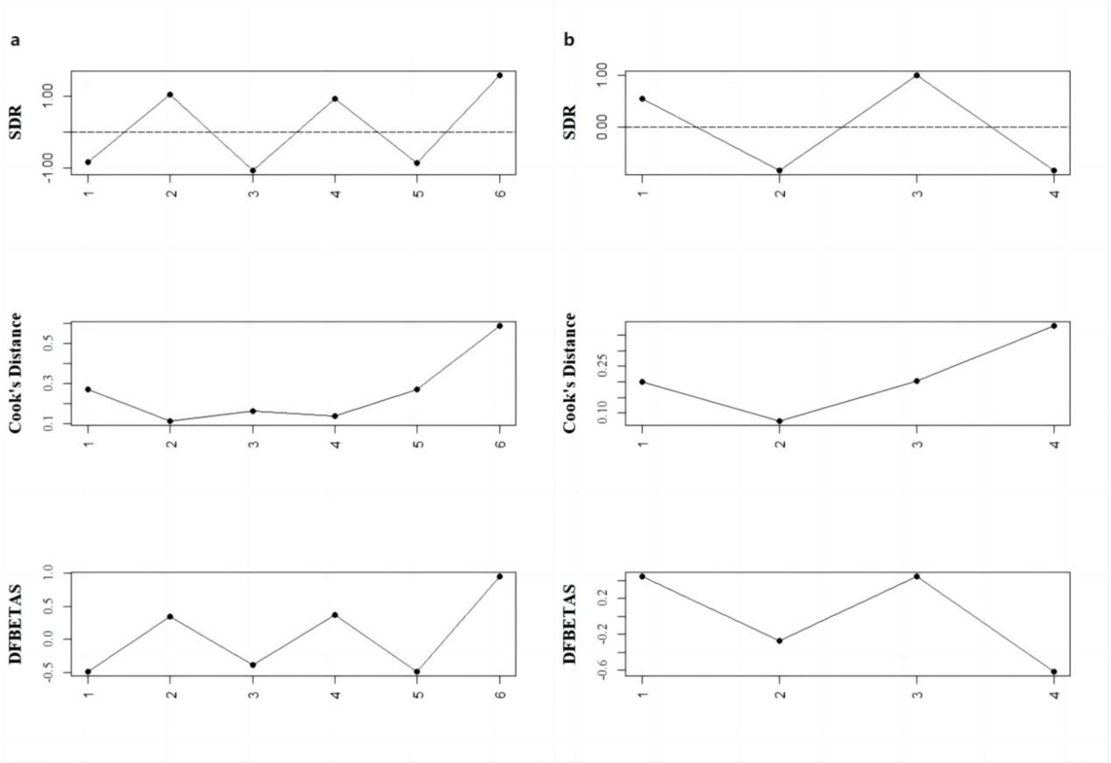

a:Outlier and influence analysis for Quality of life at post-treatment.

b: Outlier and influence analysis for Quality of life at follow-up.

Appendix F Funnel plot

Figure F1. The funnel plot for BDD symptoms at post-intervention

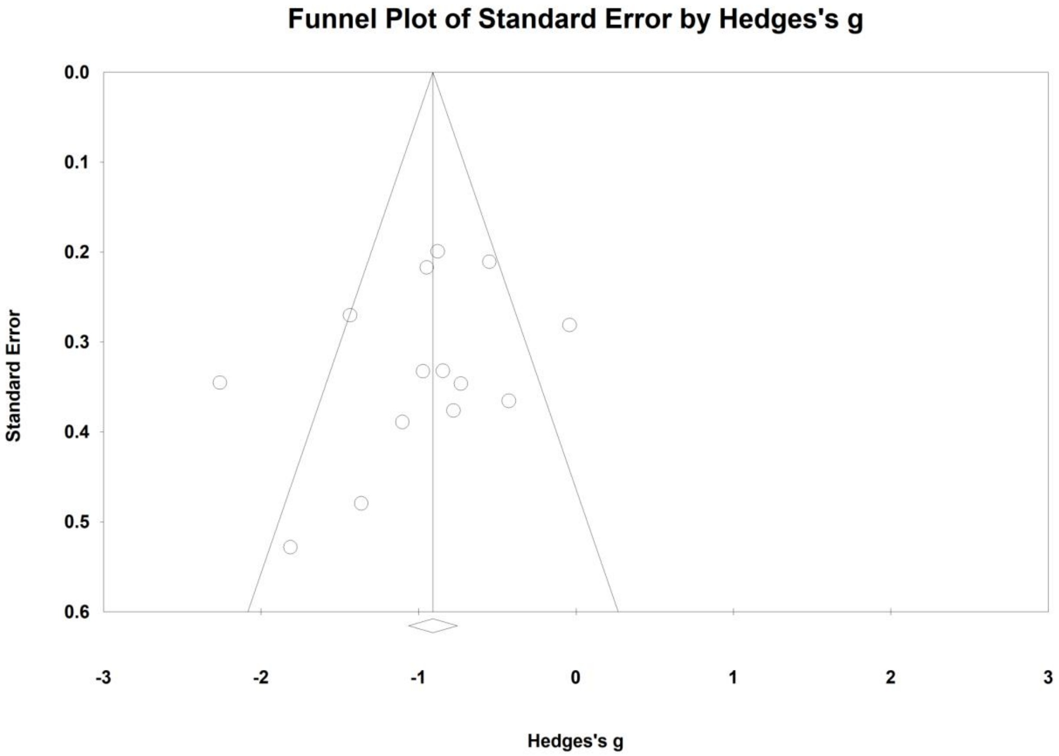

Figure F2. The funnel plot for BDD symptoms at follow-up

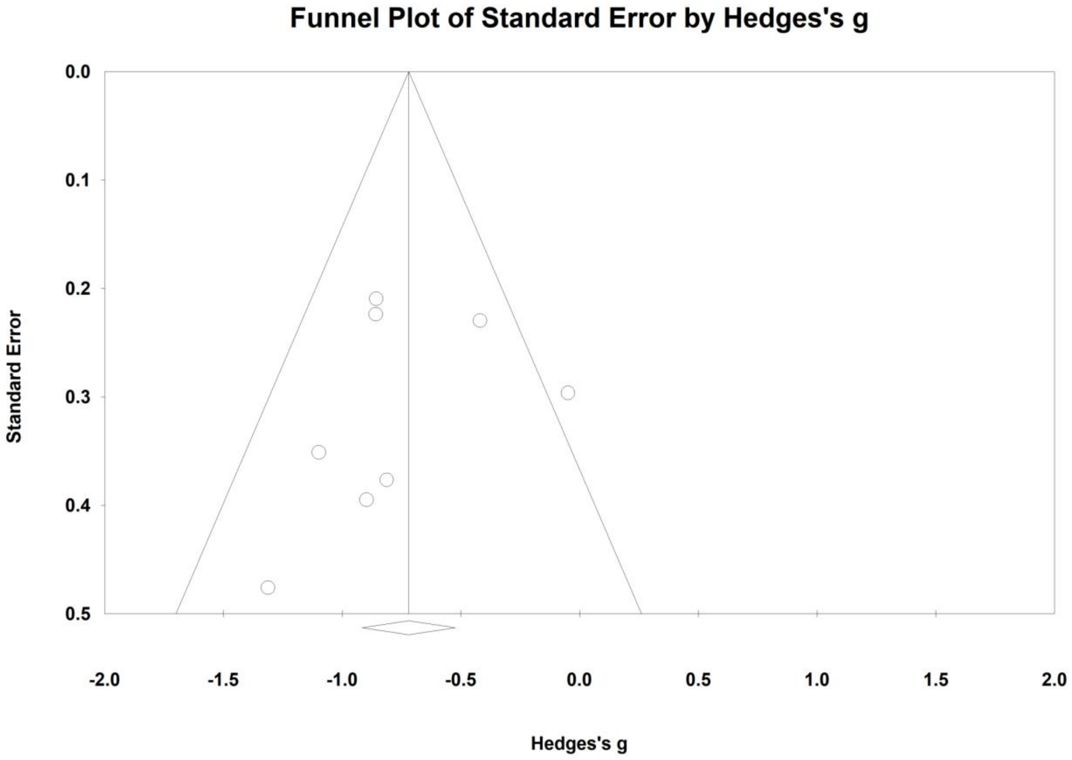

Figure F3. The funnel plot for depression symptoms at post-intervention

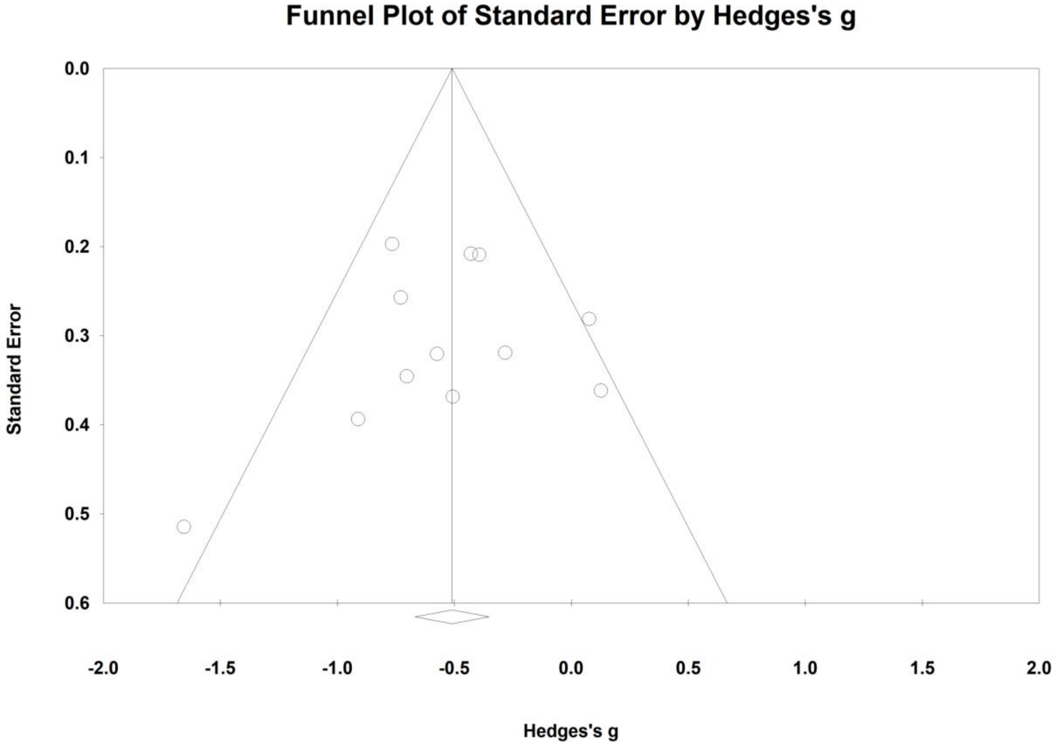

Figure F4. The funnel plot for depression symptoms at follow-up

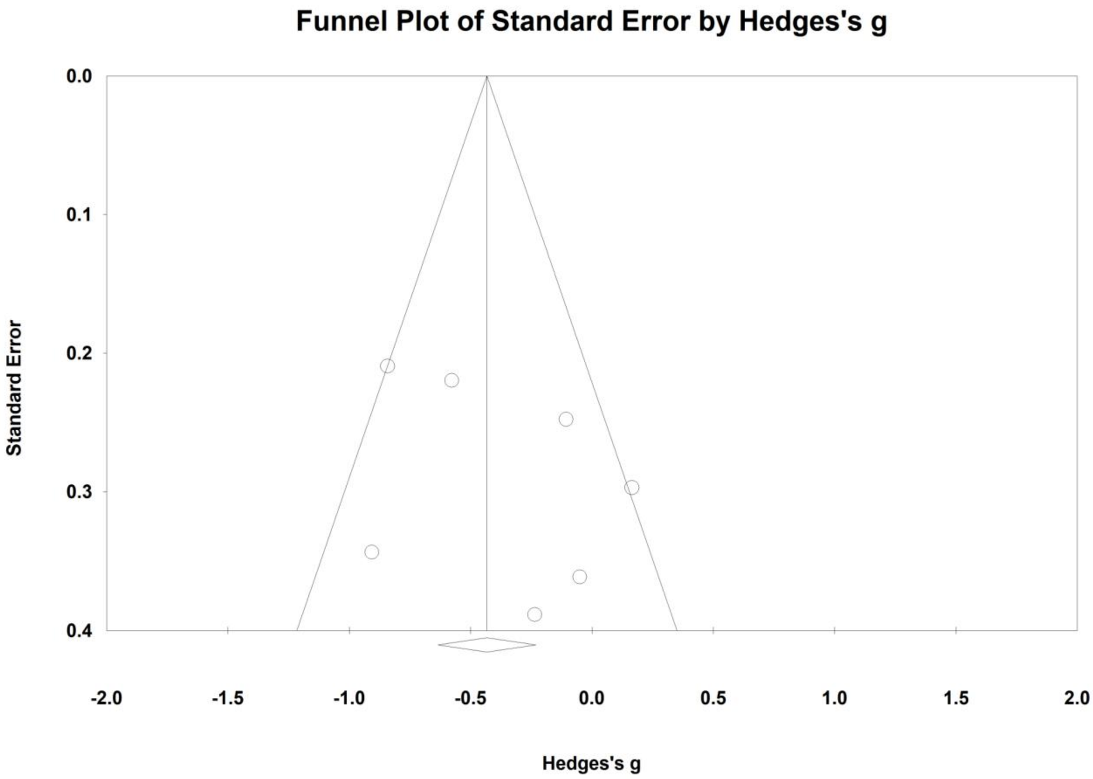

Figure F5. The funnel plot for insight at post-intervention

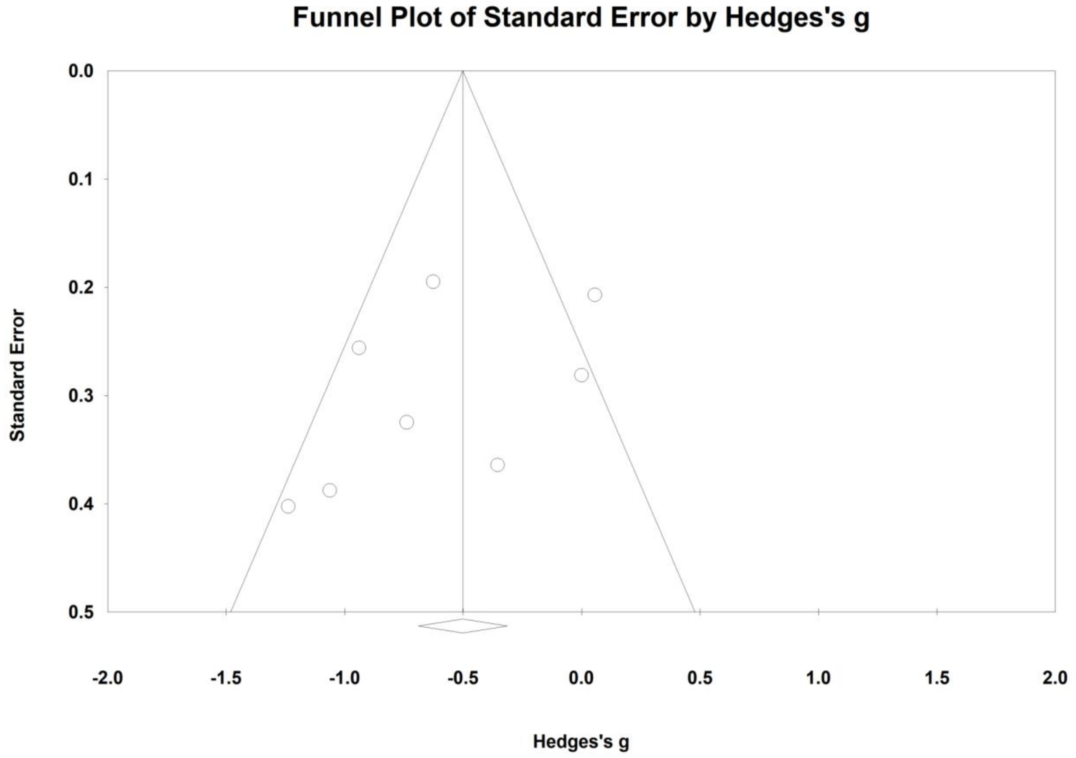

Figure F6. The funnel plot for insight at follow-up

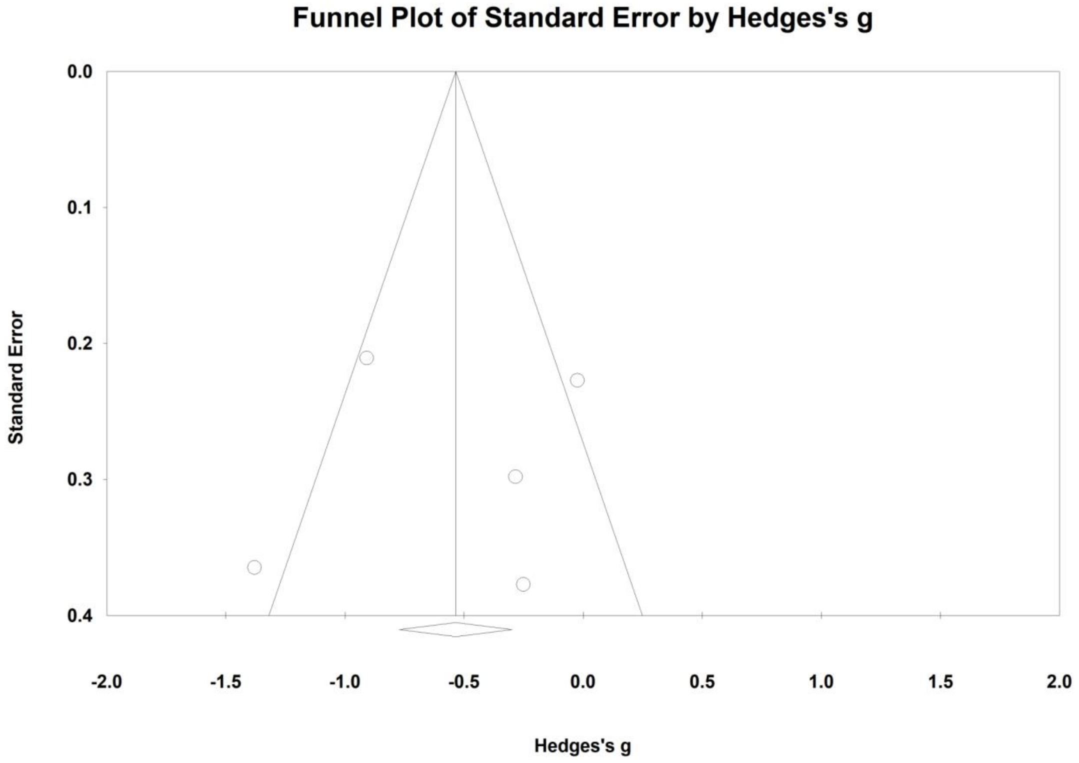

Figure F7. The funnel plot for Psychosocial functioning at post-intervention

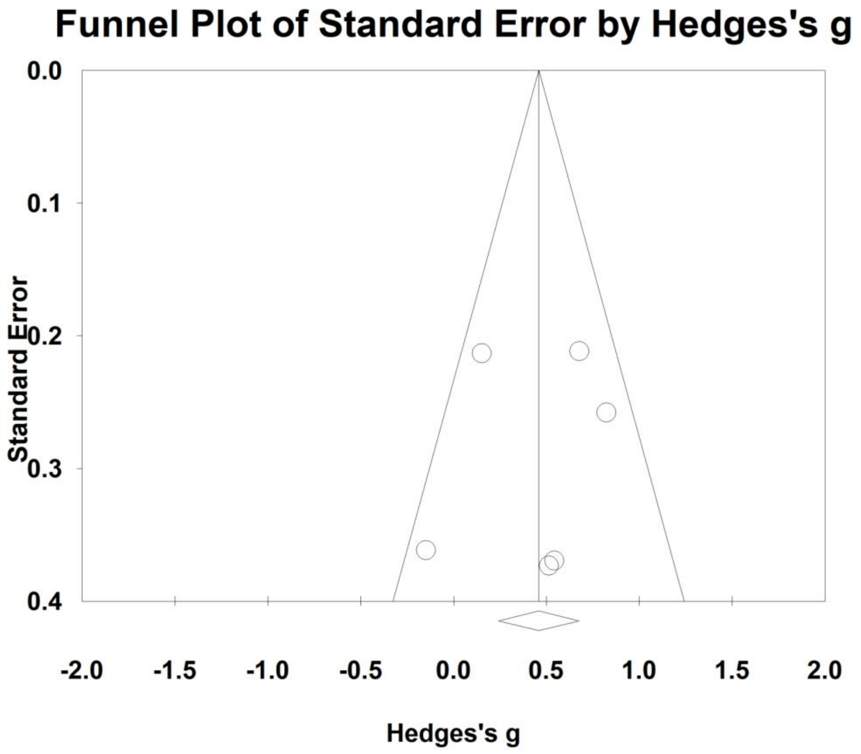

Figure F8. The funnel plot for Psychosocial functioning at follow-up

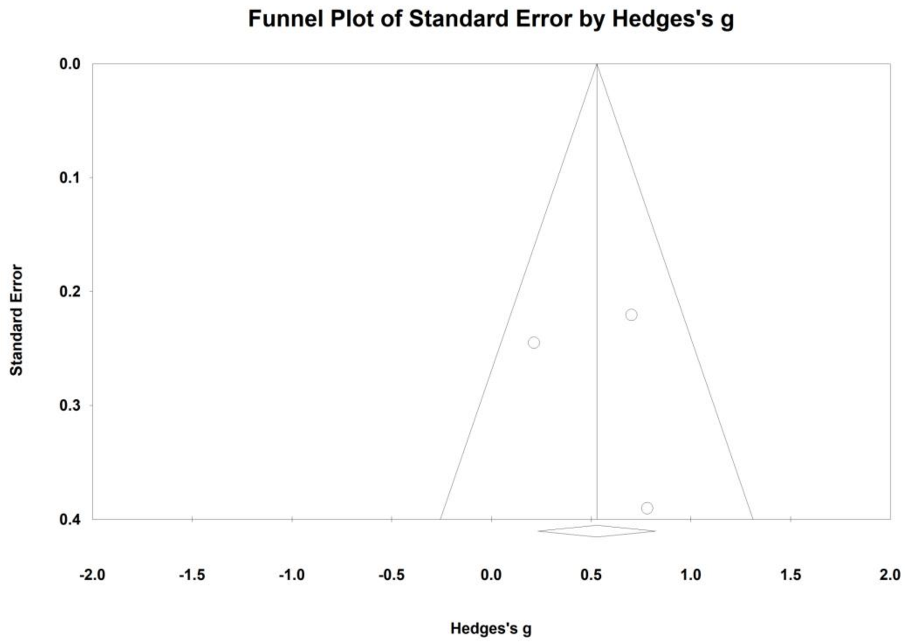

Figure F9. The funnel plot for quality of life at post-intervention

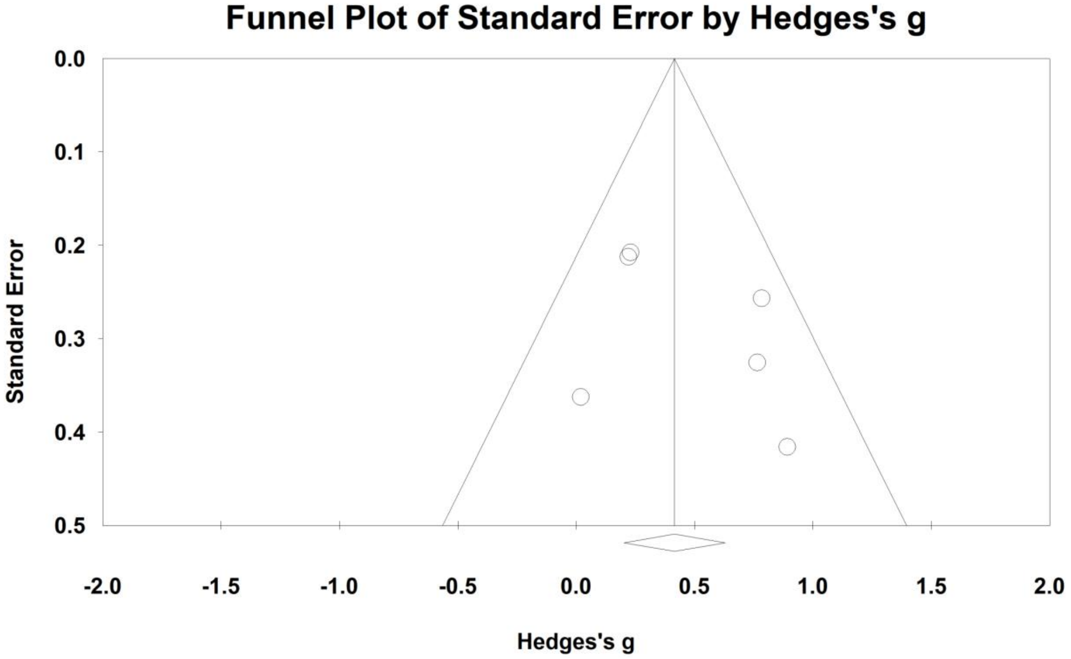

Figure F10. The funnel plot for quality of life at follow-up

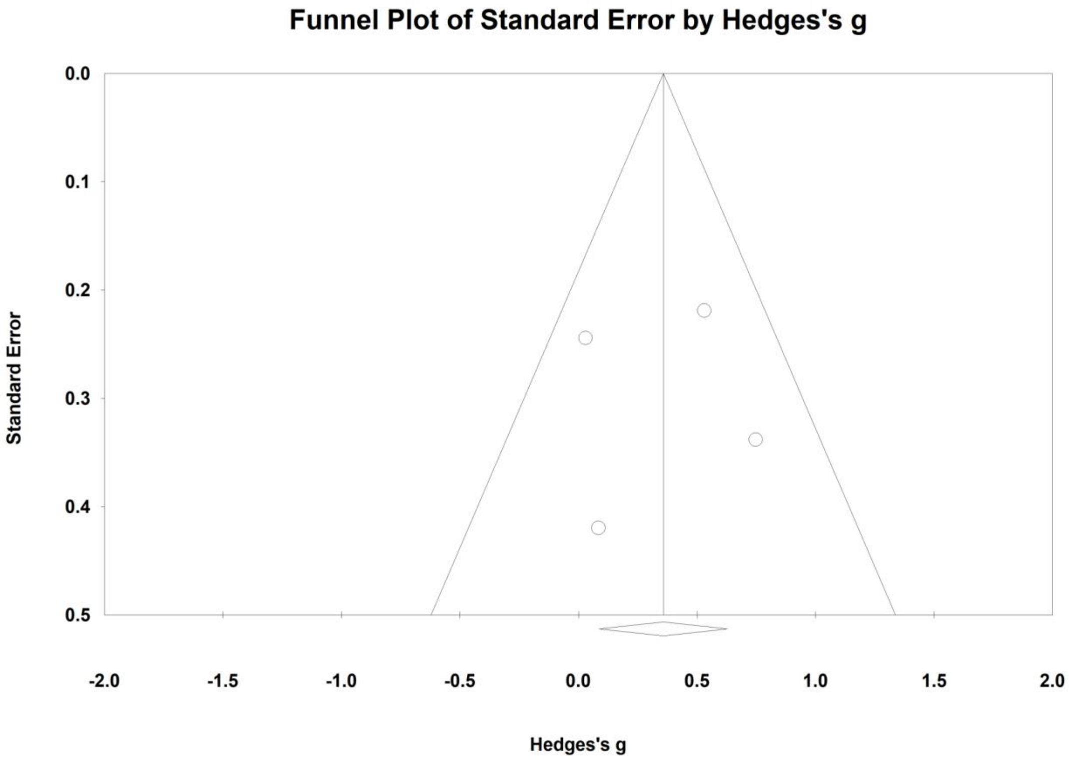

Figure F11. The funnel plot for anxiety at post-intervention

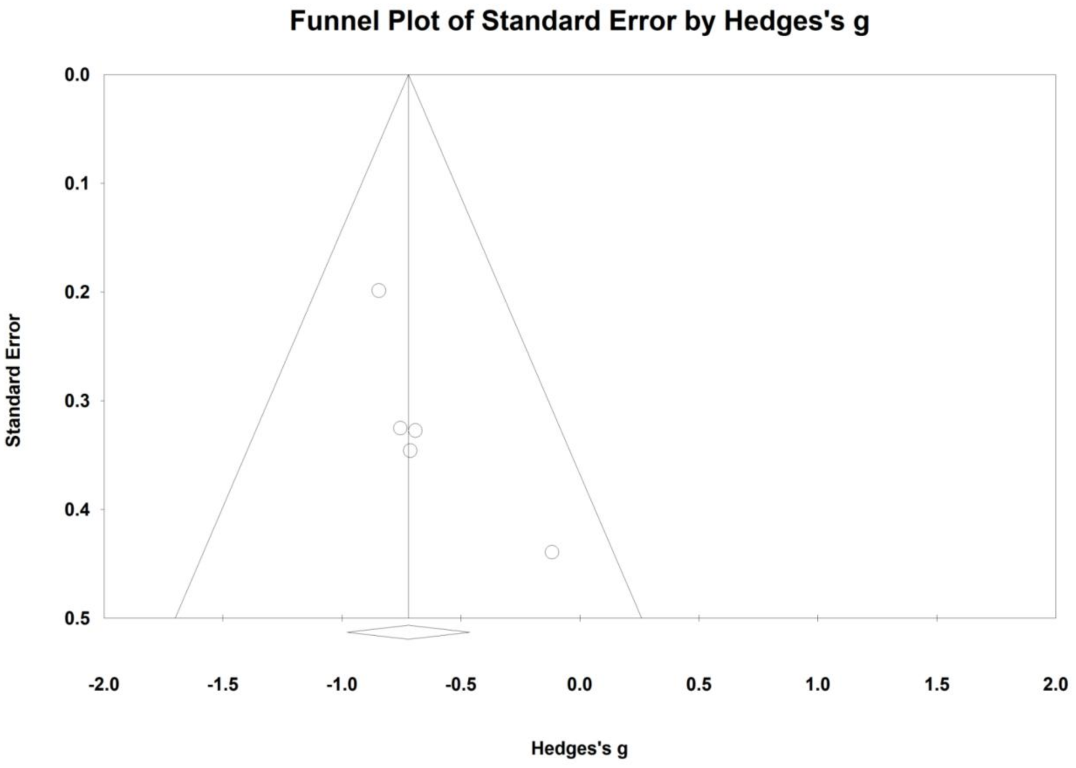

Figure F12. The funnel plot for anxiety at post-intervention

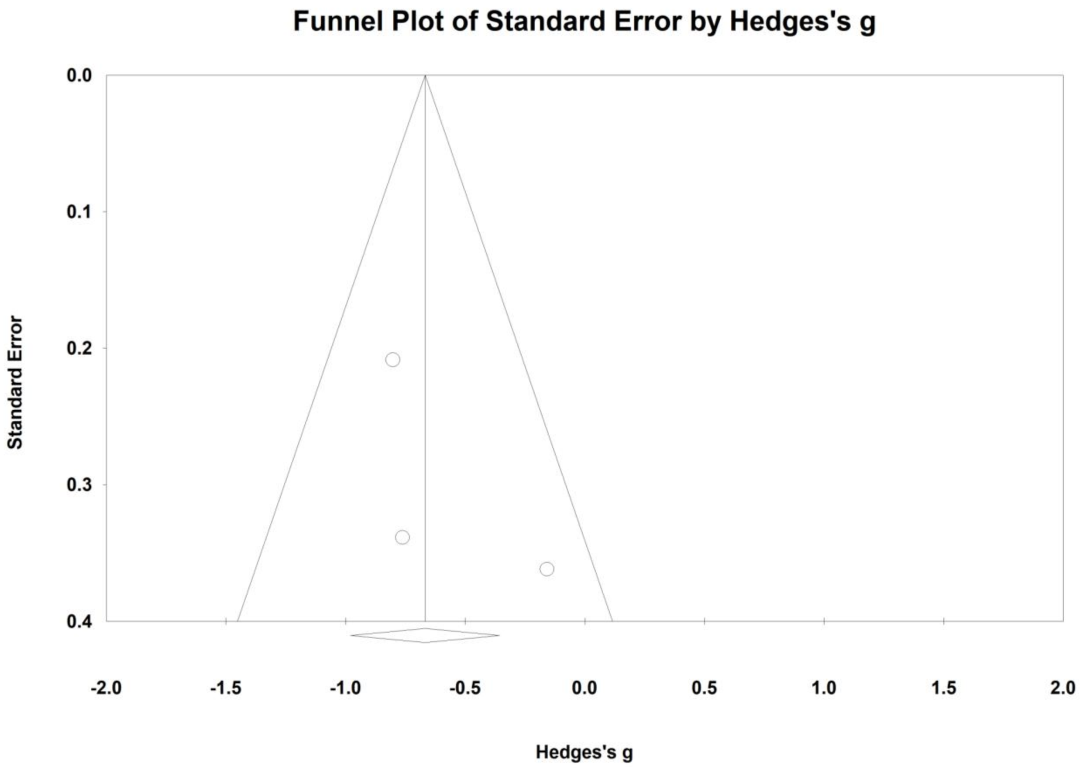

Appendix G. Trial sequence analyses

Figure G1. TSA for BDD Symptoms at post-treatment and at follow-up

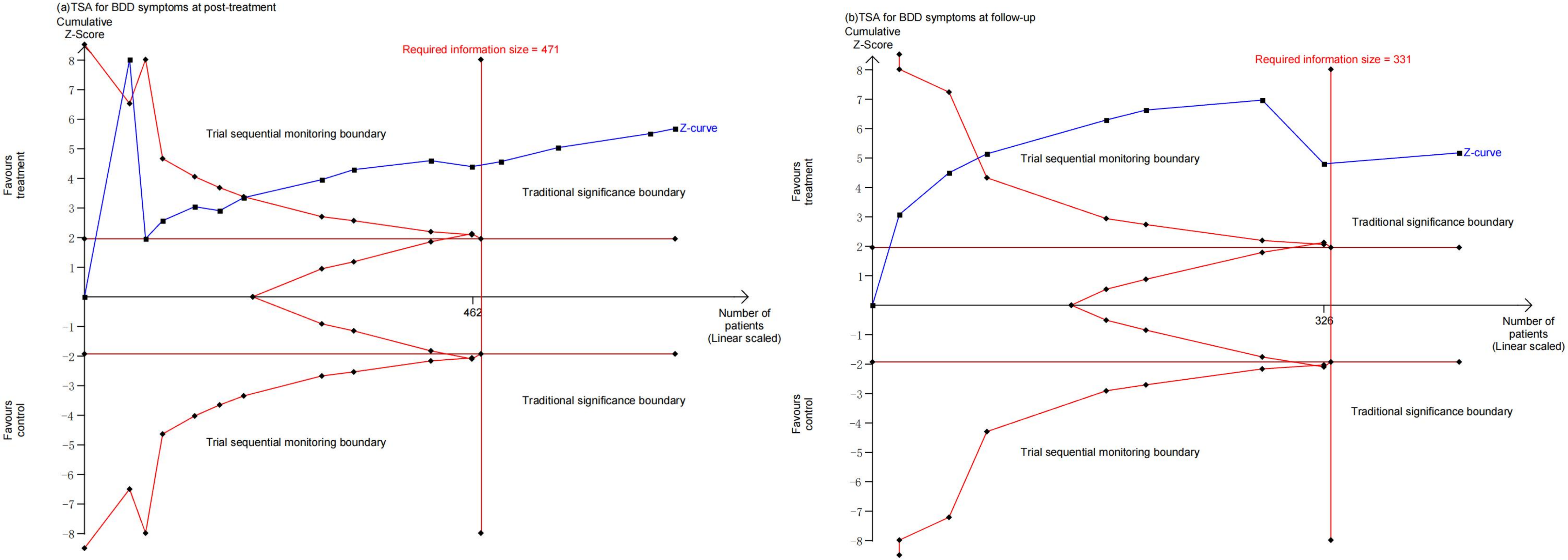

Note. On the x-axis, the total number of participants is displayed. On the y-axis, the Z values represent the statistical summary of the data. The horizontal red lines denote the conventional significance thresholds at a constant Z value of 1.96, which corresponds to a p value of 0.05. The red curved line represent the adjusted thresholds for statistical significance with an α of 0.05. The blue line is the cumulative Z curve and represents the accumulating amount of information as trials are added, each square denoting an individual trial. The vertical red line represents the required information size (sample size) as indicated by RIS.

Figure G2. TSA for depression at post-treatment and at follow-up

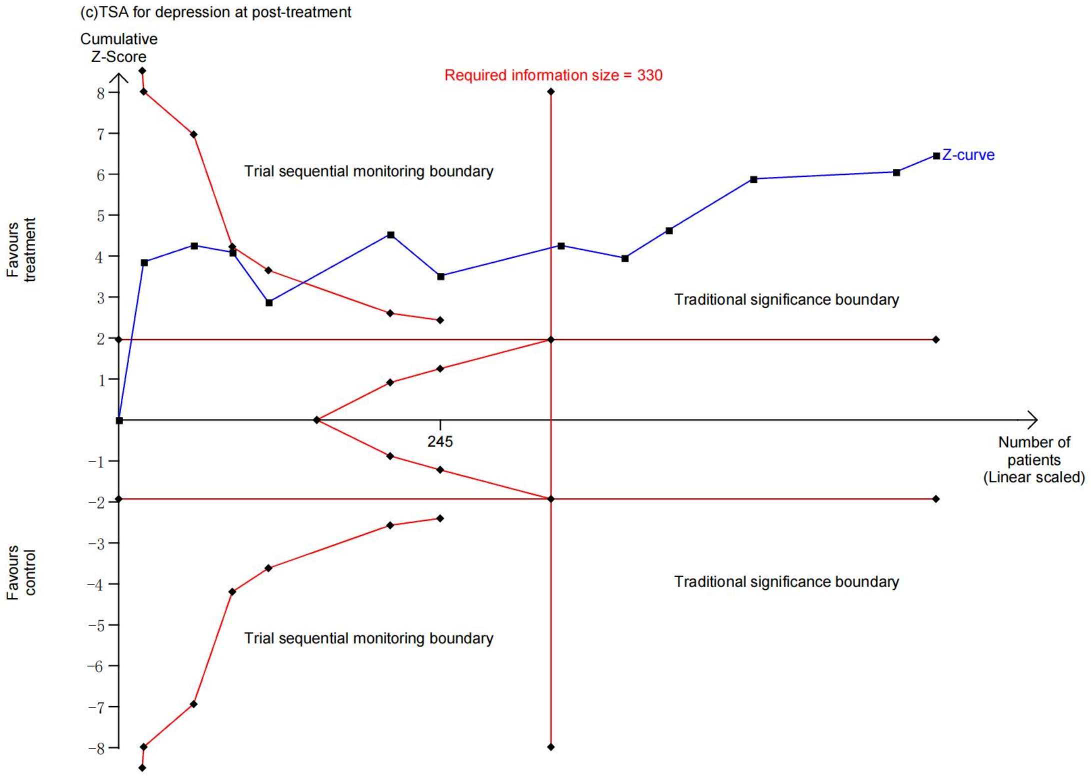

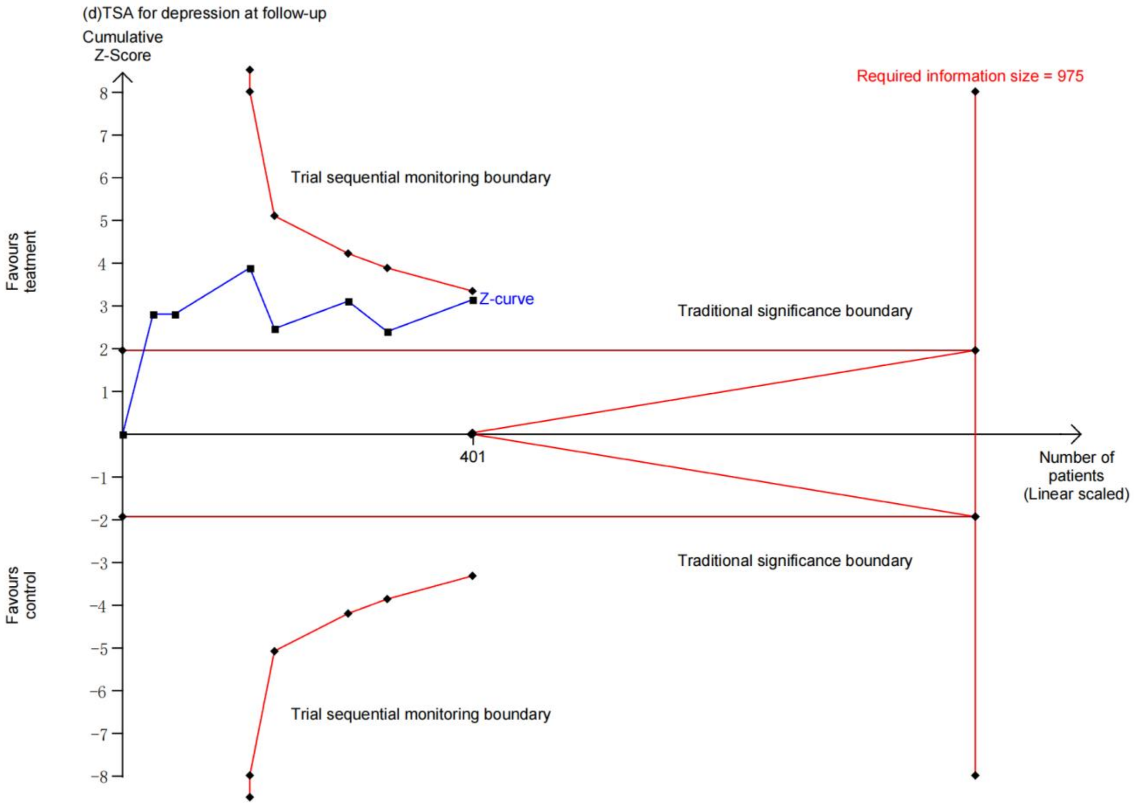

Figure G3. TSA for insight at post-treatment and at follow-up

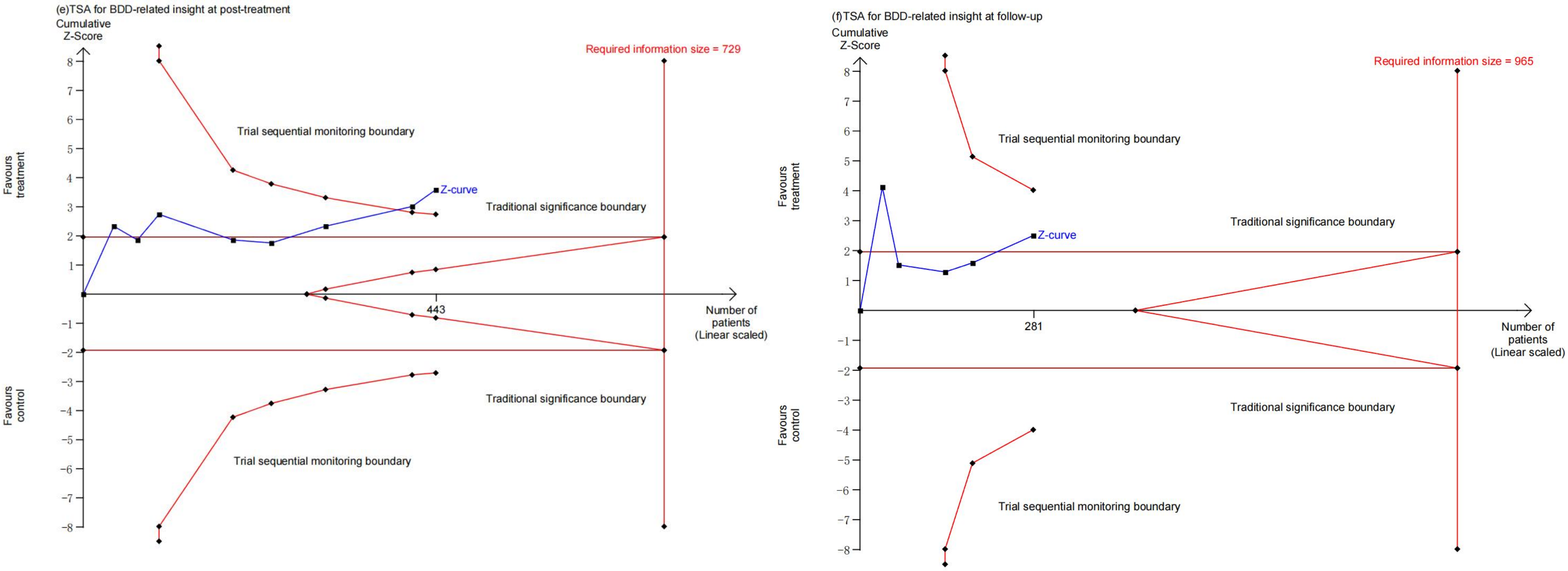

Figure G4. TSA for psychosocial functioning at post-treatment and at follow-up

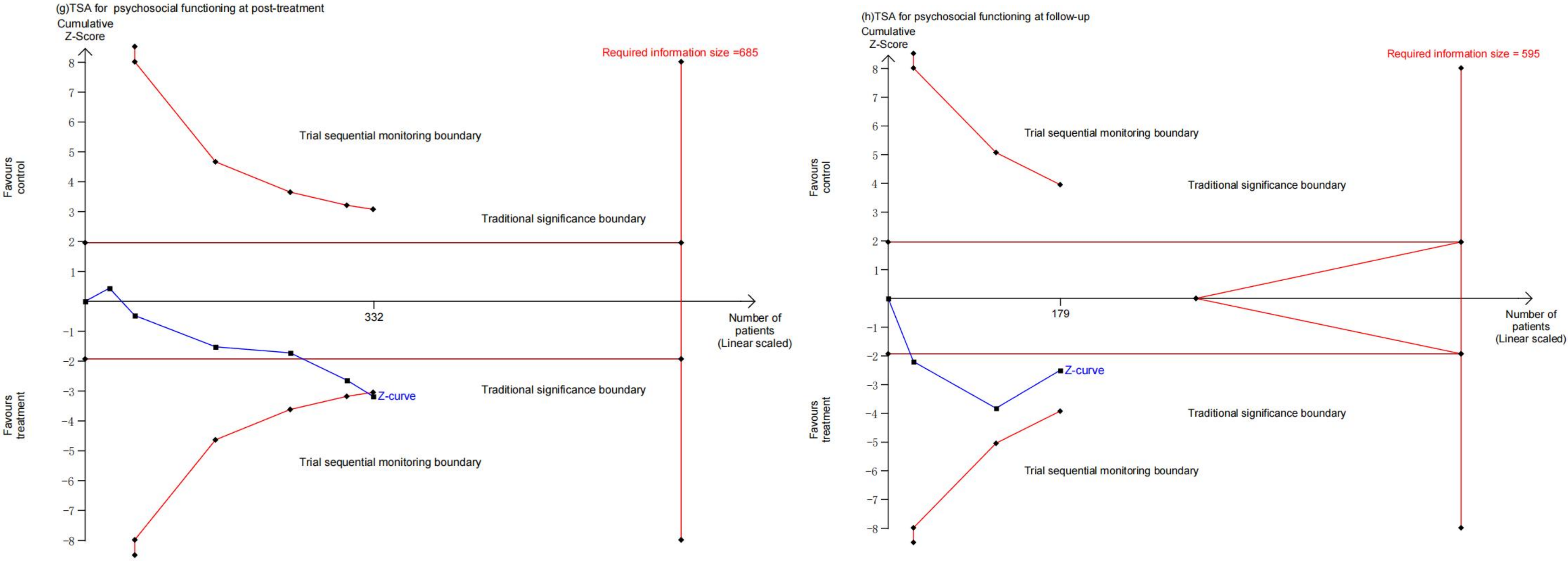

Figure G5. TSA for quality of life at post-treatment and at follow-up

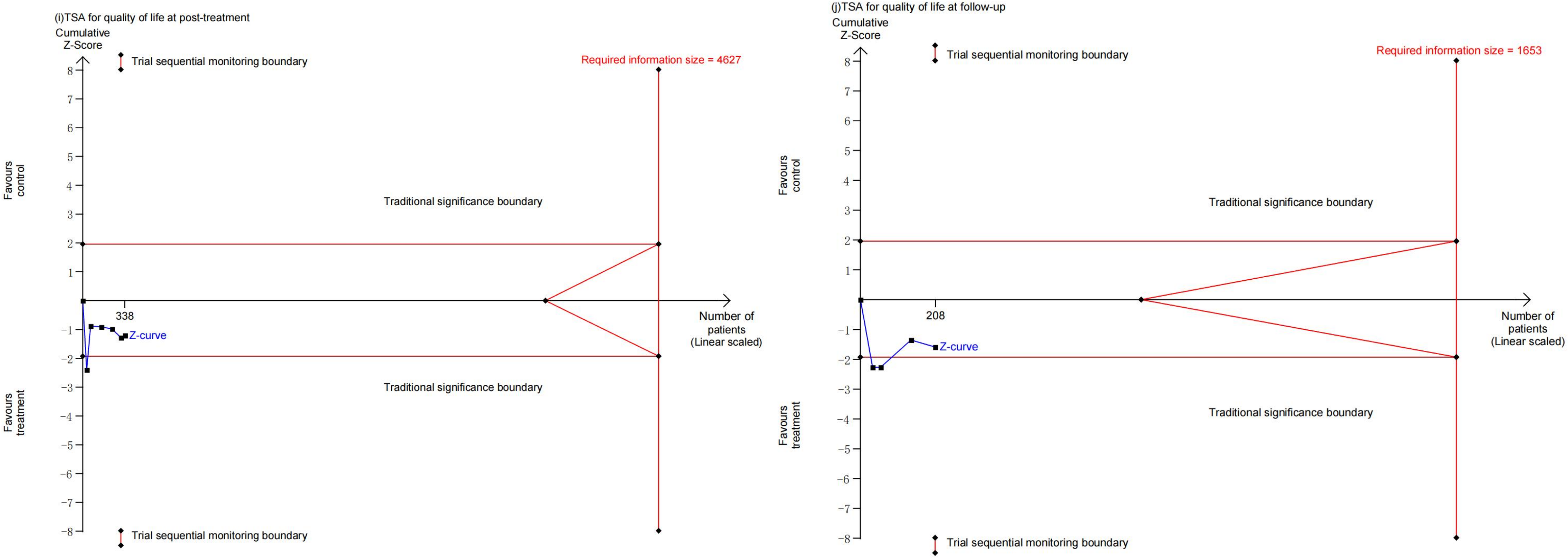

Figure G6. TSA for anxiety at post-treatment and at follow-up

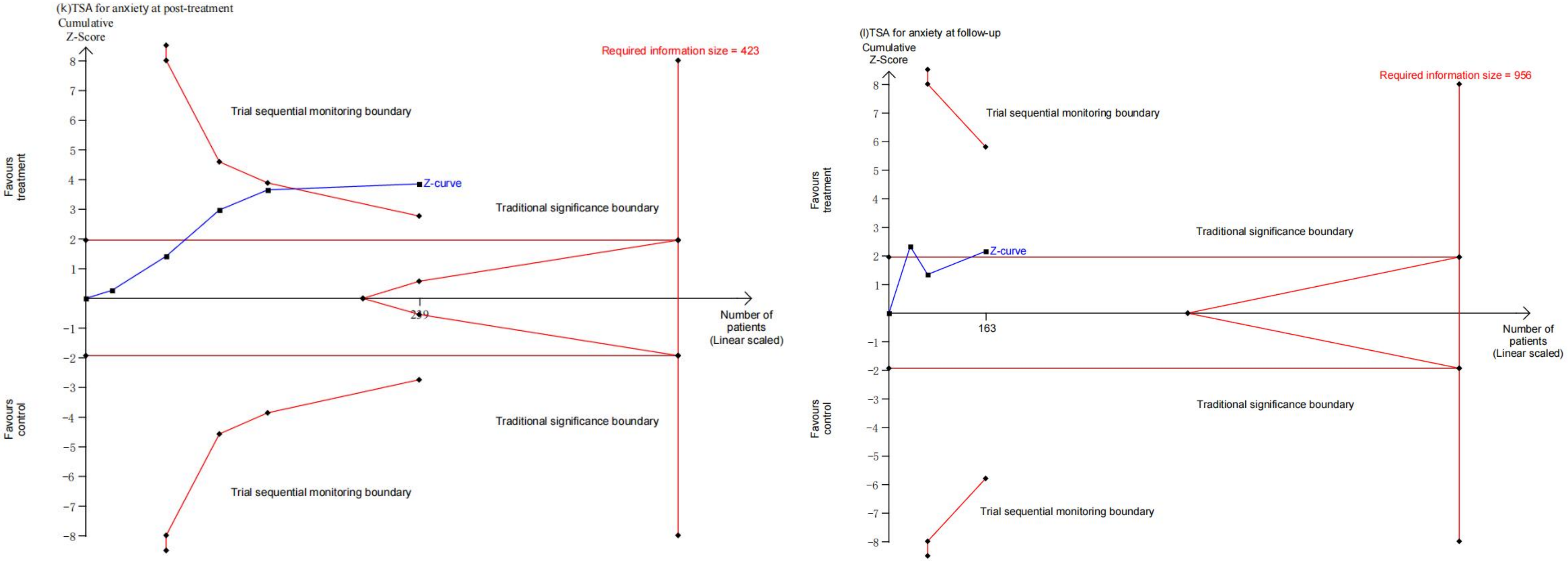

Supplement: Liu et al. supplementary material [file S0033291724002733sup001.pdf]
